# Mechanical Activation of Forbidden Photoreactivity in Oxa-di-π-methane Rearrangement

**DOI:** 10.1021/acs.joc.2c00720

**Published:** 2022-09-27

**Authors:** Alejandro Jodra, Cristina García-Iriepa, Luis Manuel Frutos

**Affiliations:** † Universidad de Alcalá, Departamento de Química Analítica, Química Física e Ingeniería Química, Grupo de Reactividad y Estructura Molecular (RESMOL), Alcalá de Henares 28806, Madrid, Spain; ‡ Instituto de Investigación Química ‘‘Andrés M. del Río’’ (IQAR), Universidad de Alcalá, Alcalá de Henares 28806, Madrid, Spain

## Abstract

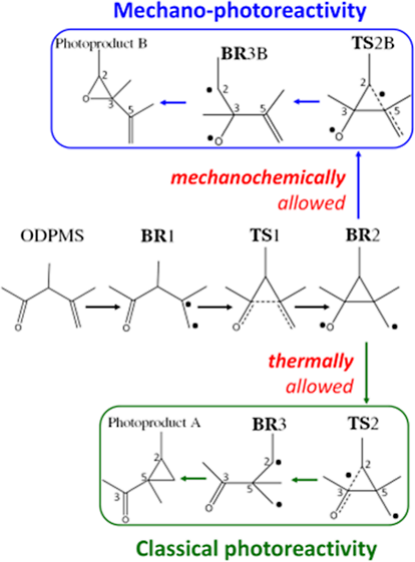

In this work, we demonstrate that the forbidden oxirane-type photoproduct (the cyclopropyl ketone photoproduct is the allowed one) of the oxa-di-π-methane photorearrangement can be obtained by mechanochemical control of the photoreactions. This control is achieved by the application of simple force pairs rationally chosen. By analyzing in detail the effect of the applied forces on this photoreaction, it comes to light that the mechanical action affects the diverse properties of the oxa-di-π-methane rearrangement, modifying all the steps of the reaction: (i) the initial ground-state conformers’ distribution becomes affected; (ii) the new conformational population makes the triplet excitation process to be changed, responding to the magnitude of the applied force; (iii) the stability of the different intermediates along the triplet pathway also becomes affected, changing the dynamical behavior of the system and the reaction kinetics; and (iv) the intersystem crossing also becomes strongly affected, making the forbidden oxirane-type photoproduct to decay more efficiently to the ground state. All these changes provide a complex scenario where a detailed study of the effect of applied forces is necessary in order to predict its overall effect on the photoreactivity.

## Introduction

The application of external forces, known as mechanochemistry, has been used in the last decade as an efficient tool to modify the reactivity and properties of given molecules.^1−3^ Experimentally, the external force can be applied through force probes (such as alkyl chains or photoswitches),^4,5^ sonication,^6−8^ or atomic force microscopy.^9,10^ Mechanochemistry has found diverse applications from force sensors to mechanobiology, chemical synthesis, or material science.^11−15^ One of the advantages of mechanochemistry is that the strength of the applied force can be modulated, through chemical design or stretching force. The application of the external force has a direct influence on the potential energy surface (PES) as the mechanical work developed by the external force is added to the potential energy of the system. This fact could influence the stationary points (minima or TS) and/or relative stability of different configurations.

Mechanochemistry can be applied to both thermal and photochemical processes. The modulation of thermal processes by external forces has been widely studied, covering thermal stability, chemical kinetics, or reactivity (activation energies and reaction products).^4,16−18^ Regarding photochemical processes, works aimed on the mechanochemical modulation of the photophysical properties (absorption or emission spectra),^19−22^ quantum yield of the process,^23,24^ or conical intersection topology^25^ have been reported. Nevertheless, there is no reported evidence that photoproducts can be changed by mechanical means in any photochemical reaction.

**Scheme 1:**
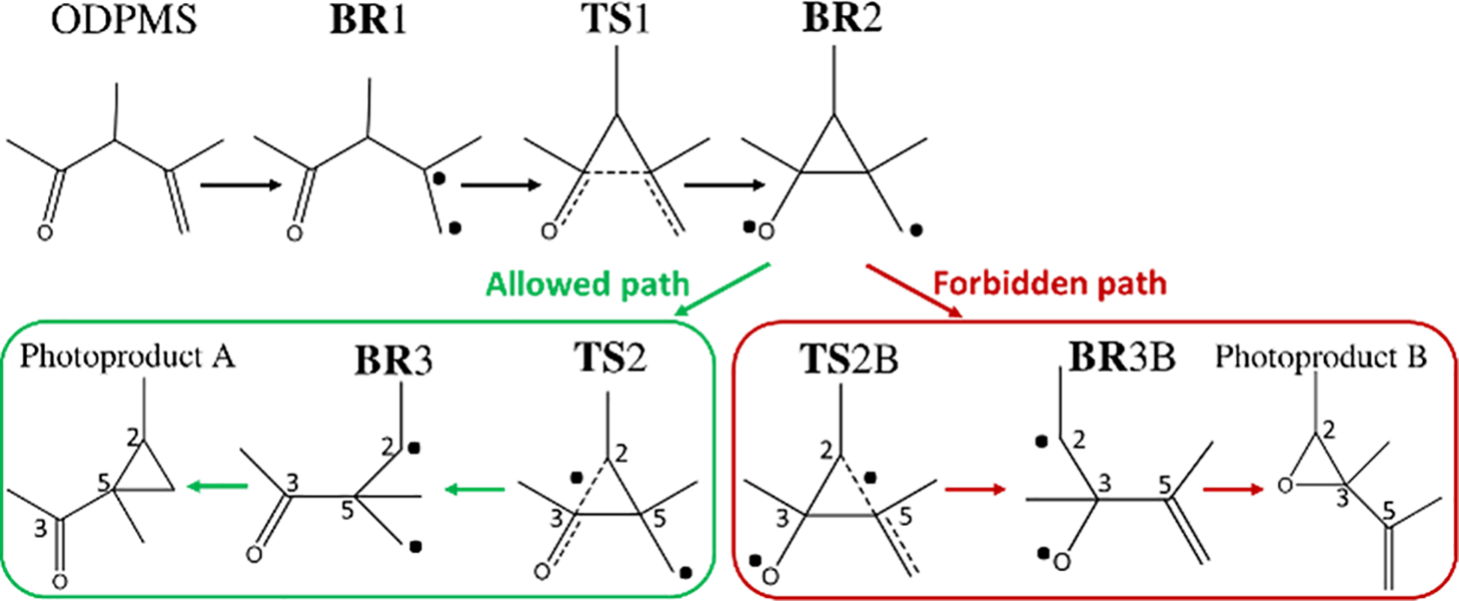
Reaction Mechanism of Photosensitized ODPM Photorearrangement

Reviewing the previous works focusing on mechanochemistry (for both thermal and photochemical reactions), it can be concluded that general trends of the effect on the mechanical force on the given system are difficult to obtain. In some cases, the reported works show unexpected results, revealing the complexity behind this field. For this reason, further mechanochemical studies focused on other processes and a deep analysis of the force effect are essential for the understanding of this field and the design of novel mechanochemical systems for applications. Motivated by this fact, we present a theoretical and computational investigation on the mechanochemical activation of a forbidden path along the well-known oxa-di-π-methane (ODPM) photorearrangement,^26−29^ where an ODPM structure (composed by two π-unsaturated C=C and C=O moieties connected by a sp^3^*methane*-type carbon) gives rise to a specific rearrangement (see Scheme 1). Previously, studies on the mechanochemical modulation of product formation on thermally activated reactions have been reported. However, in this case, the studied process is a photochemical reaction activated after photon absorption. Although it would seem that the mechanochemical study of thermally or photochemically forbidden product activation requires similar methodologies and considerations, it is true only to a certain extent. Analyzing the mechanical effect on the photochemical photoproduct activation demands challenging studies compared to the thermal processes as: (i) the effect of the applied force may be significantly different on the ground than on the excited states; (ii) the crossings between electronic states (e.g., conical intersections or avoided crossings) will be in general affected by mechanical forces provoking eventually a change in the photochemical pathways; and (iii) the usual short lifetime of excited-state species makes the effect of applied forces on these states more complex to predict as molecular dynamics is usually necessary to correctly describe this effect.

Coming back to the studied system, ODPM, its photorearrangement was first reported in 1966;^30^ since then, it has been widely used for synthetic purposes, for natural products, or complex molecules.^31−34^ The general mechanism of the ODPM photorearrangement consists of a first 1,2-acyl migration and a subsequent cyclization (Scheme 1), the formation of two photoproducts (photoproduct A and B in Scheme 1) being possible in principle. In practice, it has been experimentally found that the path leading to the cyclopropyl ketone (photoproduct A in Scheme 1) is allowed, whereas the one leading to the oxirane photoproduct (photoproduct B in Scheme 1) is forbidden.^26^ For a better understanding of the overall mechanism in terms of the involved electronic states, it can be summarized on a first ground-state (S_0_) photosensitization, leading to the population of the first triplet state (T_1_). Once in T_1_, the system evolves populating different biradical intermediates (see Scheme 1), hopping back to S_0_ by intersystem crossing and forming the final photoproducts.

It should be remarked that no previous mechanochemical studies of the activation of a forbidden photoproduct have been reported, implying the already mentioned challenging issues compared to thermal product activation. For this aim, we have selected, by chemical intuition, a force pair that should have the largest effect in favoring the oxirane instead of cyclopropyl ketone product. Once the applied force is selected, we analyze its effect on the PESs in terms of: (i) ground-state conformational equilibrium, (ii) the sensitized excitation to the triplet state; (iii) the evolution through different intermediates on T_1_; and (iv) the decay to S_0_, leading to photoproduct formation. By analyzing these results, we show that the mechanochemical activation of the forbidden oxirane photoproduct is possible in ODPM. However, behind this general conclusion, remarkable findings on this mechanical control are hidden. For instance, the applied forces affect the kinetics on T_1_, making the forbidden photoproduct preferred in thermodynamic control conditions. Additionally, the kinetic control becomes also strongly affected by the applied force, as the spin–orbit coupling term strongly increases with the applied force in the case of the forbidden photoproduct, making this photoproduct feasible under mechanical control.

## Methodology

### Electronic Structure Methods

Ground-state (S_0_) and first T_1_ stationary points of ODPM have been optimized using density functional theory (DFT) methodology. Specifically, we have chosen the B3LYP functional^35,36^ and the 6-31G(d) basis set, validated by a benchmark with the available theoretical data (see the Supporting Information for details). Analytical energy gradients and Hessians were calculated at the same level of theory. Frequency calculations were performed to characterize minima and transition states. Single point energy calculations have been performed using the complete active space self-consistent field (CASSCF) theory for selected DFT-optimized geometries. In particular, an active space of eight electrons in six orbitals and the 6-31G(d) basis set have been used. These calculations were performed with the Gaussian09^37^ suite of programs. Spin orbit coupling values have been computed at the DFT level of theory (using the same functional and basis set as that for the optimization) with ORCA 4.2.1.^38,39^

### Inclusion of External Forces to the System

The external force type considered in this work consists of a force pair applied to two anchor points, as shown in Figure 2. These external forces have been explicitly included in the force field (therefore in the PESs) for exploring the PES and computing molecular dynamics when necessary. In the specific case of stationary points, the constrained geometries simulate external force approach has been used, which is equivalent in these cases to the explicit inclusion of the external forces in the force field (see the Supporting Information for further details).

### Triplet Energy Excitation Distribution

Taking into account the ground-sate equilibrium population of all the conformers, the triplet excitation distribution has been constructed as a convolution of the distribution of each conformer, weighted by its equilibrium population. This procedure has been followed for any considered external force (see the Supporting Information for further details).

### Dynamics

The dynamic behavior of the strained system at different force strengths has been studied at the DFT level of theory (B3LYP/6-31G(d)) by performing constant energy (i.e., microcanonical ensemble) dynamics. All the trajectories (T_1_ state) have been computed by sampling (*T* = 300 K) the initial state (i.e., ground-state minimum in the first set of dynamics and **BR**2 in the second one) (see the Supporting Information for sampling details). These dynamics are performed numerically using our own code, based on gradient calculations performed by the Gaussian09 suite of programs and explicitly adding, when necessary, the external forces to the force field (see the Supporting Information for more details).

### Reaction Pathway Considering the External Force

The determination of reaction paths for systems affected by external forces has been performed by adding the mechanical work developed by the applied forces to the potential energy. This work corresponds to the usual work path integral and corrects the normal PES by including the mechanical work term.

## Results

The overall mechanism has been summarized in the Introduction section. In brief, T_1_ state is populated after photosensitization. In this triplet state, diverse biradical intermediates are generated until decay to S_0_ takes place, leading the photoproducts. The obtained results are presented in two main sections: (i) the detailed mechanism of the unstrained ODPM photorearrangement and (ii) analysis of the mechanical control of the forbidden photoproduct activation. In particular, the second section is in turn divided, considering the properties that the mechanical force can affect: (i) ground-state conformational equilibrium, (ii) triplet excitation energy, (iii) triplet excitation process and excitation migration, (iv) T_1_ reaction pathway, and (v) ground-state photoproduct formation.

### Unstrained ODPM Photorearrangement

**Figure 1:**
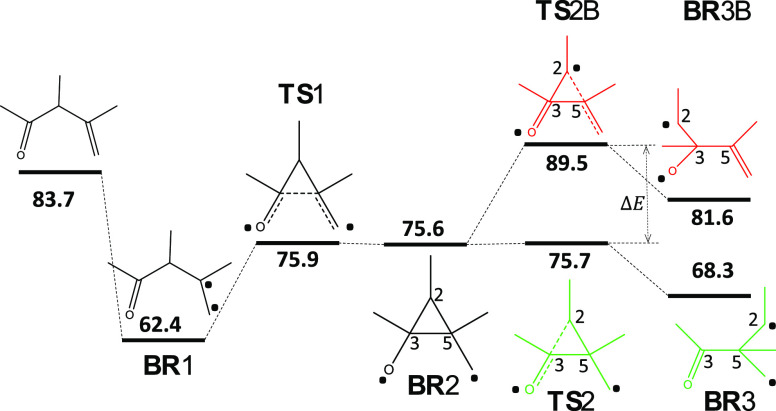
Reaction pathway in T_1_ (B3LYP/6-31G(d) energies in kcal/mol, referenced to the S_0_ minimum energy of the most stable conformer E. After triplet excitation, ODPM could evolve through different biradical species: **BR**1 and **BR**2. From **BR**2, the reaction pathway bifurcates, the formation of **BR**3 being possible through the transition-state **TS**2 and **BR**3B through **TS**2B. **BR**3 corresponds to the allowed photochemical pathway, while **BR**3B to the forbidden one. Atom numbering is depicted as well as the radical localization for each species by a dot point (located in the atoms having the largest spin density).

By analyzing in detail the T_1_ path, we can conclude that two photoproducts would be feasible by considering the possible radical pairs formed (Scheme 1). Specifically, the C3–C5 bond breaking of **BR**2 may lead to the formation of two different biradicals: **BR**3 (formed through **TS**2) and **BR**3B (through **TS**2B). Analyzing the computed energies (at the DFT level), referenced to the S_0_ minimum, we can conclude that the formation of **BR**3 is kinetically favored as **TS**2B implies a ca. 14 kcal/mol higher energy barrier than **TS**2(Figure 1). Moreover, the formation of this biradical is also thermodynamically driven as it is ca. 13 kcal/mol more stable than **BR**3B (Figure 1). This makes the formation of photoproduct B forbidden, and in fact, its formation through the ODPM photorearrangement mechanism has neither been reported nor experimentally detected.

### Mechanical Control of the Forbidden Photoproduct Activation

**Figure 2:**
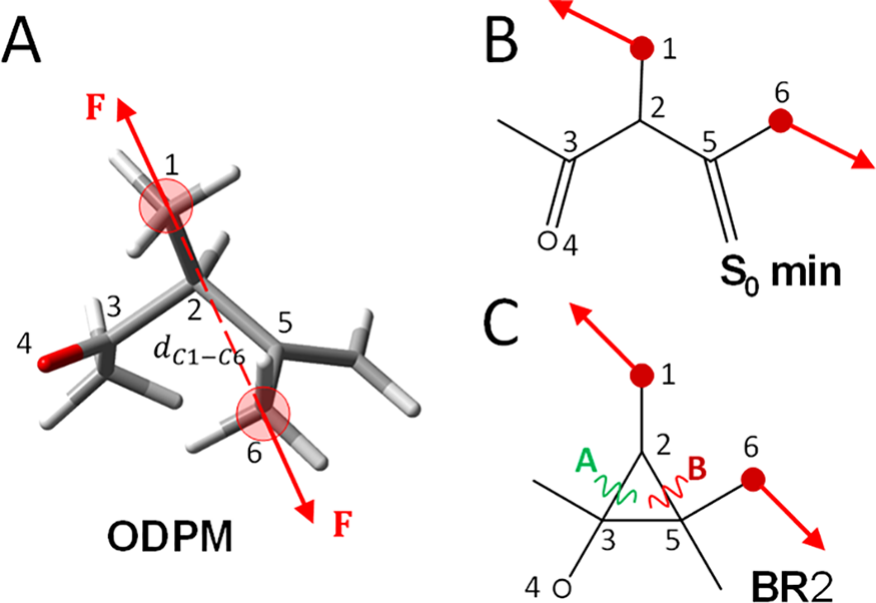
(A) ODPM starting structure showing numbering, and the applied force pair depicted as vectors and defined as the distance between atoms C1 and C6 (d_C1–C6_). (B) Schematic view of the S_0_ energy minimum structure. (C) **BR**2 structures indicating the applied force pair. The possible photoproducts are formed by breaking the bond A (photochemical allowed path) or B (photochemical forbidden path) from **BR**2 species. Red dots indicate the atoms where the force is applied.

Mechanochemical control of the photoreaction may be feasible if the applied external force has a relevant component along the reaction coordinate.^24,25^ This situation makes the reaction to be mechanically affected and may alter the thermochemical parameters and kinetics of the process.^16^ In the case that two reaction pathways are competing, the applied force should have a differential component along each pathway in order to control the relative efficiency of each pathway, that is, it has to affect each pathway in a significantly different extent by performing different works along each reaction coordinate. For ODPM photorearrangement, the two pathways bifurcate on **BR**2 species in T_1_, being possible to access **TS**2 (allowed mechanism, green path in Scheme 1 and Figure 1) or **TS**2B (forbidden mechanism, red path in Scheme 1 and Figure 1). The main difference between both pathways is the specific C2–C3 or C2–C5 bond breaking to yield the formation of **BR**3 and **BR**3B, respectively (Figure 1 for numbering). According to this, applying an external force pair directly to C2 and C5 atoms may affect the relative energy of the involved species (transition states as well as biradical minima). In fact, the application of an extension force to the C2–C5 bond would stabilize the pathway where this bond is broken, the forbidden path. This stabilization is the result of the work developed by the applied force along the reaction path. Hence, by applying this type of external forces, it is expected to increase the feasibility of the forbidden pathway with respect to the allowed path through mechanochemistry. Experimentally, external forces are usually applied indirectly, for example*,* through molecular force probes.^4,23,40^ In order to mimic this approach, we have introduced methyl substituents in positions 2 and 5, to make possible the application of the external force pair directly to these methyl groups (C1–C6 carbon atoms, see Figure 2), inducing a similar mechanical effect that could be achieved with molecular force probes or polymer mechanochemistry.

In order to investigate the mechanical response, it is necessary to explicitly include the external forces in the force field and analyze their effect on the reaction mechanism. In the following, different aspects of this mechanophotoreaction, ODPM photorearrangement where a C1–C6 force pair is applied, are studied: i) the ground-sate conformational equilibrium, ii) the triplet excitation energy, iii) triplet excitation process and excitation migration, iv) evolution on T_1_, and v) photoproduct formation in S_0_.

### Ground-State Conformational Equilibrium

**Figure 3:**
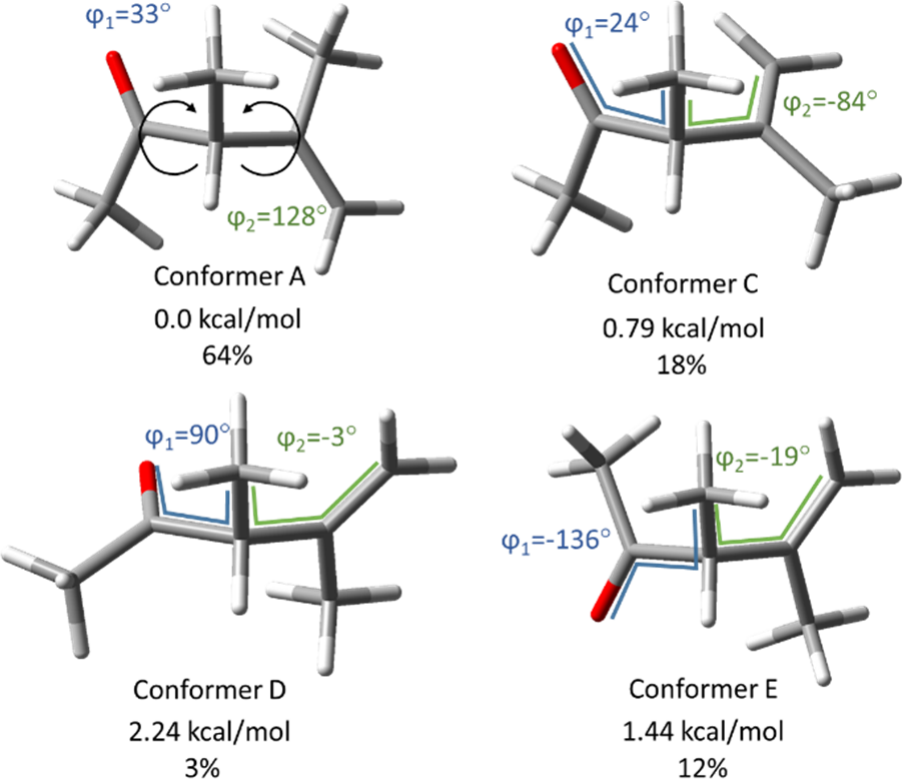
Structure and relative energy (computed at the DFT level) of the four most populated conformers of ODPM (**A**, **C**, **D**, and **E**) in S_0_. The equilibrium population (in percentage) of each conformer at 300 K in the absence of external forces is also indicated.

Due to the flexibility of the ODPM system, there are different conformations that can be populated in S_0_. More specifically, a total of eight conformers (**A**, **B**, **C**, **D**, **E**, **F**, **G,** and **H**; see Figures 3 and S2) related by the value of the two main torsional angles (i.e., φ_1_ and φ_2_, as defined in Figure 3) can be populated.

**Figure 4:**
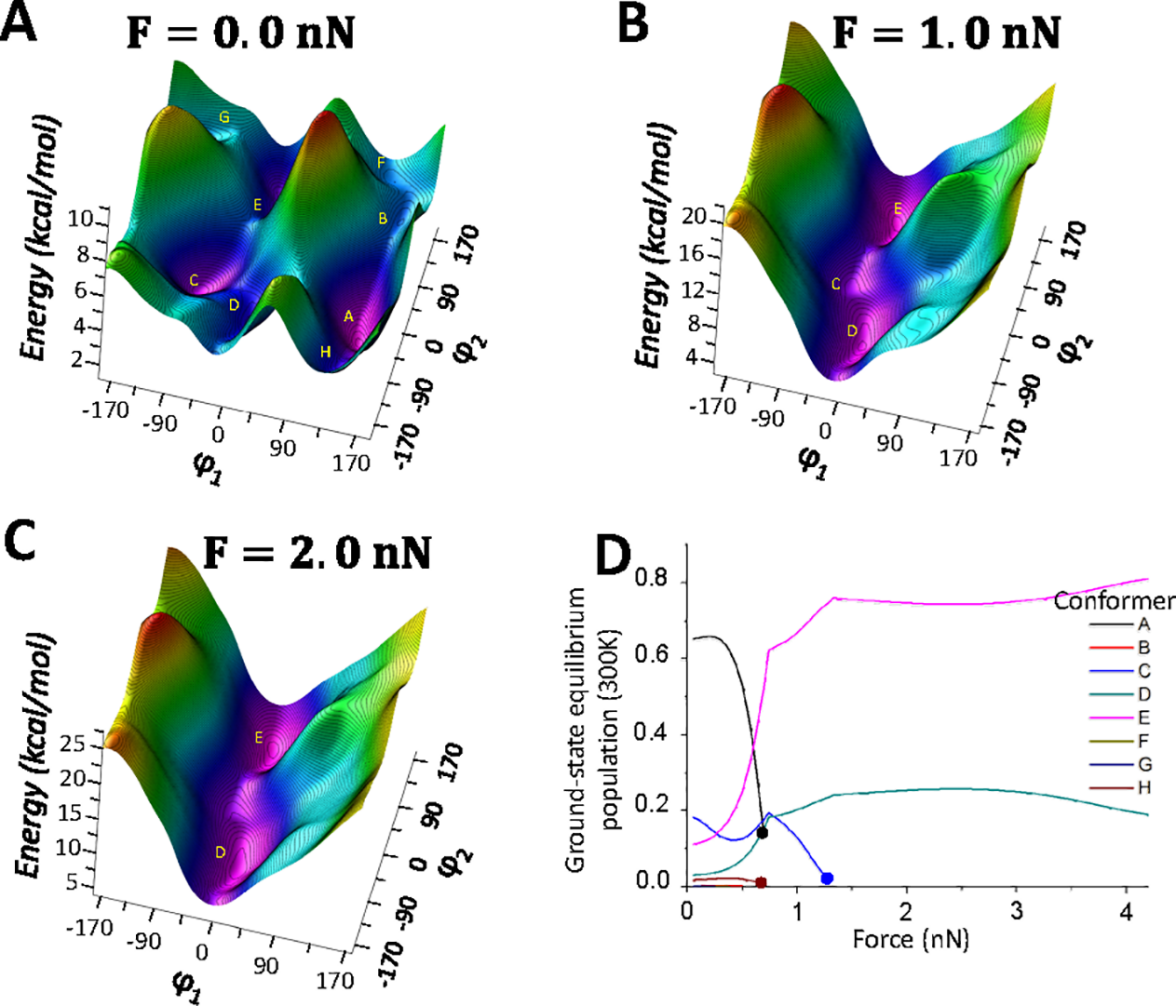
(A–C) S_0_ PESs as a function of φ_1_ and φ_2_ (in degrees) coordinates for different applied forces (0.0, 1.0, and 2.0 nN). Conformer minima are indicated by their corresponding labels. (D) Equilibrium population for the different conformers (**A**–**H**) at 300 K for the applied force magnitudes ranging from 0 to 4 nN). Dots indicate the forces at which the conformers **A**, **C,** and **H** disappear (i.e., no longer minima on the PES).

In the absence of external forces, conformers **A**, **C,** and **E** are the most abundant in thermal equilibrium conditions (see Figure 4D). Applying the already described external force pair (defined by the C1–C6 distance, d_C1–C6_ in Figure 2), the conformational equilibrium is modified (changing the population of each conformer) to different extent, depending on the force magnitude (see Figure 4). By analyzing the population of each conformer regarding the applied force magnitude, we can remark three findings. First, conformers **A** and **C**, which are the two most populated conformers for the unstrained system̧ are not any more minima for external force magnitudes of ca. 0.7 and 1.3 nN, respectively (Figure 4D). This fact is the result of the progressive destabilization of a minimum on the PES as the force magnitude increases, making the curvature of the PES moving from positive along specific coordinates to negative. When the curvature becomes exactly zero, the minimum on the PES disappears (see Figure 4A–C). The second observation is of crucial importance, as for force magnitudes larger than 0.5 nN, conformer **A** is no longer the most populated one, but the **E** conformer is (Figure 4D). This information has to be considered to properly study the force effect on the mechanism. Finally, the third finding is that some conformers such as **C** and **D** also became relevant for higher force magnitude ranges (see Figure 4B–D), being responsible for ca. 20% of the population at different force magnitudes.

### Triplet Excitation Energy

**Figure 5:**
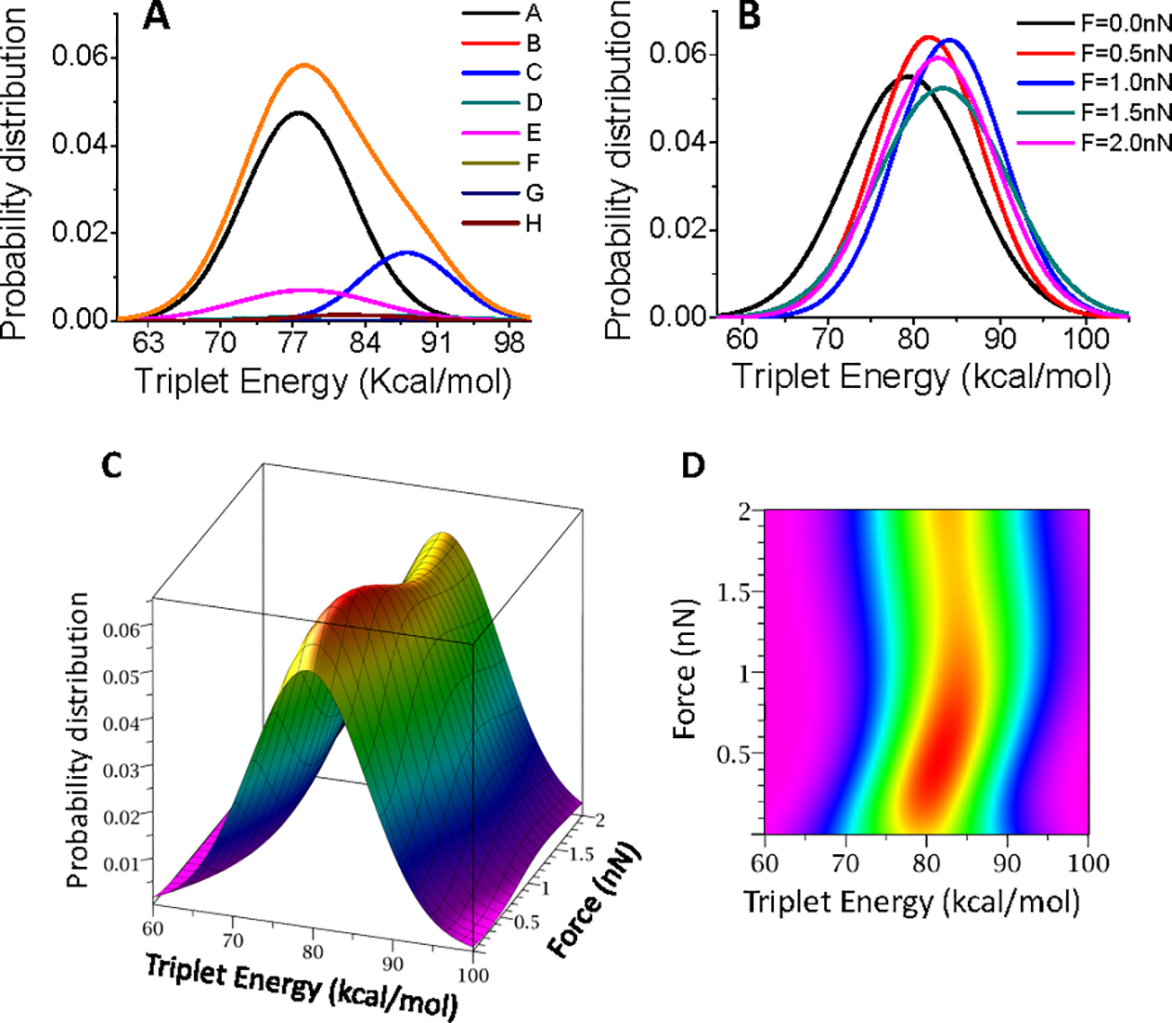
Probability distribution of the triplet excitation energy in the ODPM system. (A) Distribution probability for the unstrained system of each conformer weighted by its abundance at 300 K. The resulting sum is also plotted (orange curve). (B) Total (summation over all conformers at 300 K) triplet energy distribution at different forces (from 0 to 2 nN). (C) Continuous triplet energy probability distribution as a function of the force strength and (D) corresponding contour map (up view).

The photochemical reaction is initiated by a photosensitizer, that is, the triplet energy is transferred from the photosensitizer to the reacting ODPM molecule. The triplet energy transfer takes place when there is a match between the transferred energy (from the photosensitizer) and the triplet energy of the accepting system (i.e., S_0_–T_1_ energy gap of the reacting ODPM system). Otherwise, the triplet energy transfer rate constant decays exponentially.^41^ The observed ODPM triplet excitation energy is the collective contribution of the different conformers and their populations. The application of an external force may affect the observed triplet excitation energy because not only the triplet excitation energy of each conformer but also its population will be affected, as already shown. In this regard, we have studied the individual triplet energies of each conformer at different applied force magnitudes. Summing up all the individual contributions and weighting them by its equilibrium populations, it is possible to predict the probability distribution of triplet excitation energy at any force magnitude (see the Supporting Information for details). This probability distribution provides the relative probability of triplet excitation as a function of the triplet energy of the donor analogously, as the absorption spectrum in an optical excitation provides the relative probability of excitation as a function of the energy photon (e.g., wavelength). In the absence of external forces, the most probable triplet energy is close to that of conformer **A** because of its high population (64%) (orange and black curves in Figure 5A). When applying C1–C6 forces, the shape of the probability distribution slightly changes, the triplet energy of maximum probability being shifted ca. 5 kcal/mol to higher values for 1 nN force (see Figure 5B). This energy shift should affect the rate constant of the photosensitization energy transfer process due to its exponential dependence with the S_0_–T_1_ energy difference. In fact, applications of this type of external forces should require the use of higher triplet energy donors in order to maintain the optimal energy transfer rate.^42,43^

### Triplet Excitation Process and Excitation Migration

As already mentioned, the ODPM photorearrangement is a photosensitized reaction. In particular, the available energy from the photosensitizer can, in principle, excite two different chromophores in the ODPM system, the C=O or C=C moiety. The photosensitizer triplet energy may determine which one is excited, as has been noted before.^42^ The lowest triplet energy state (T_1_) corresponds to the C=C moiety, as can be inferred from the spin density analysis (see the Supporting Information for further details). This excitation has been assumed to be the one taking place in this kind of photorearrangements.^26^ For the ODPM derivative studied in this work, both excitations (C=O or C=C moiety) will reach the same **BR**2 biradical.^40^ Therefore, the system loses the memory of which chromophore accepts the triplet energy. Therefore, from the experimental point of view, it is not possible to infer from the photoproducts, which excitation is taking place. Nevertheless, in the case of multichromophore systems, not studied here, the triplet-excited moiety could play a relevant role as different triplet excitations would lead to different photoproducts.

**Figure 6:**
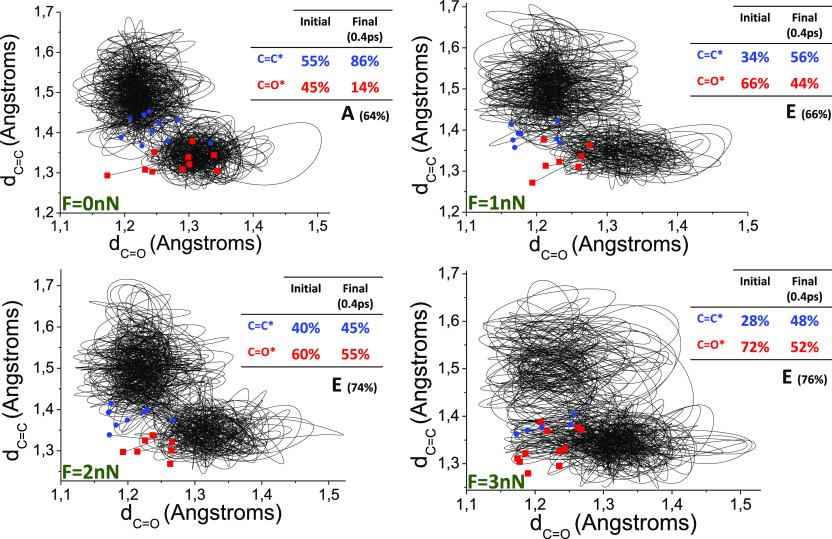
Evolution of simulated trajectories after vertical excitation to T_1_ as a function of C=C and C=O bond distances for different external forces (*F* = 0 nN, *F* = 1 nN, *F* = 2 nN, and *F* = 3 nN) corresponding to the most probable conformer (conformer **A**, for 0 nN, and **E** for other forces). Red (blue) dots indicate the vertical excitation structures for C=O (C=C) excitation (starting point of the trajectory). Trajectories reaching large C–O (or C–C) distances indicate the localization of the excitation in the C=O (or C=C) moiety. Initial and final (after 400 fs simulation on T_1_) excitation proportions are indicated in the inset table.

In this regard, we have studied the influence of the external force on the triplet excitation process and the potential migration of the triplet energy among the two chromophores (i.e., C=C and C=O). To investigate the feasibility of energy migration, a series of molecular dynamic simulations have been performed. Starting from S_0_ Boltzmann distribution at 300 K, the system is excited to T_1_ and followed the evolution on this state for ca. 400 fs. This simulation time is enough to identify the formation of **BR**1 but of course not enough to achieve further intermediates, as the formation of **BR**2 or **BR**3 is expected to take place in much larger timescales (energy barriers higher than 10 kcal/mol). Interestingly, the simulations show that, in the case of the unstrained system, the excitation probability of C=C and C=O is almost equal, that is, the S_0_ configurational distribution makes the triplet excitation energies being close to equally probable (*ca.* 55% C=C excitation and 45% C=O excitation) because of the similar triplet excitation energies determined for the S_0_ sampling structures. This finding indicates that assuming the excitation of the C=C moiety could not be correct in all the cases. However, as the force pair is applied, the triplet excitation energy distribution is affected, the excitation of the C=O moiety becoming more probable (ca. 70% for 3 nN force) (see Figure 6).

Additionally, in order to check if the intramolecular triplet energy transfer among C=C and C=O moieties in **BR**1 could be feasible, as has been proposed elsewhere,^42^ we studied the potential excitation energy migrations. In this regard, we qualitatively analyzed the excitation energy transfer among C=C and C=O moieties by measuring the number of crossings between both states. More specifically, we conducted the dynamics in such a way that the trajectory is propagated always in the lowest triplet state (i.e., triplet C=C or triplet C=O). This ensures that in every crossing event the system always hops to T_1_ and does not populate T_2_. This protocol is equivalent to force a 100% hopping probability for any crossing event, which overestimates the real energy transfer rate but provides a qualitative picture of the process. In our case, we have found that C=O to C=C energy transfer events significantly increase when external forces are applied (compare initial and final values in Figure 6), showing that state crossings are feasible in the timescale of hundreds of femtoseconds.

### T_1_ Reaction Pathway

Summarily, after populating T_1_, the ODPM system passes through different intermediates in this electronic state: **BR**1→ **BR**2 → **BR**3 (see Figure 1). **BR**3 is the key structure for decaying to S_0_ for two reasons. First, T_1_ and S_0_ energies are almost degenerated (see the Supporting Information) in **BR**3. Second, the almost perpendicular disposition of the two atomic orbitals hosting each unpaired electron makes the spin–orbit coupling to be maximum, which is a characteristic feature of the sensitized di-π-methane photorearrangement.^44^

The triplet energy pathway has been determined for different external force magnitudes (C1–C6 force pair), recalculating in each case the critical points on the PES and determining the energy profiles by including the mechanical work developed by the external force (see the Supporting Information for details).

**Figure 7:**
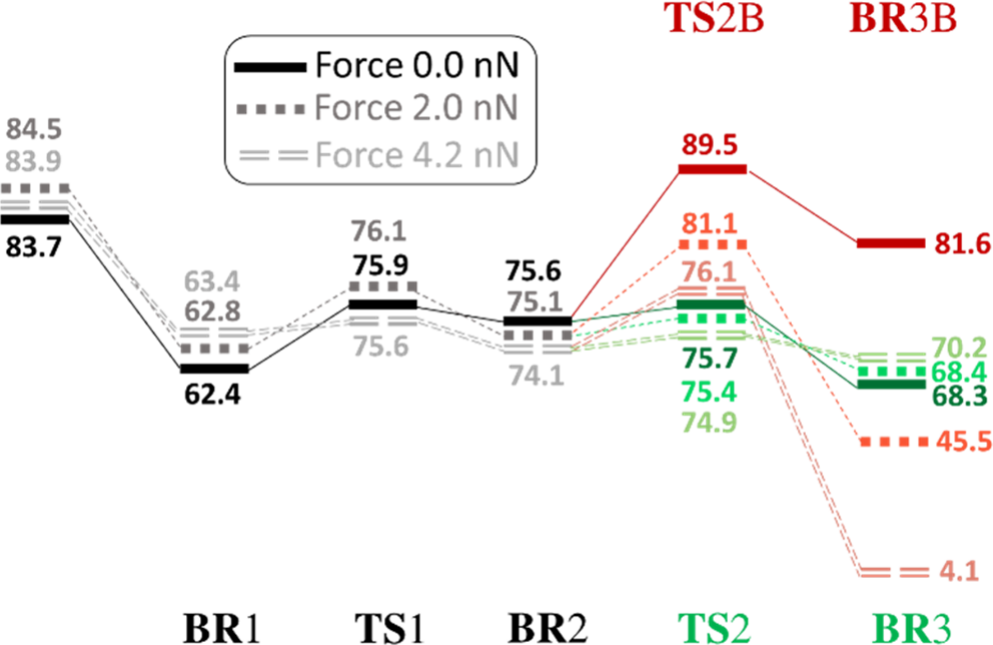
Reaction pathways (energies in kcal·mol^–1^) along the T_1_ state for three different external forces (magnitudes: 0, 2.0, and 4.2 nN) for conformer E. As it can be observed, the energy profile does not substantially change with the applied force up to **BR**2, but it significantly changes from this point.

The application of the C1–C6 external force clearly affects the reaction energy profile (see Figure 7). In fact, up to **BR**2, the energy profile does not strongly change, as expected. As a remainder, the C1–C6 force pair was chosen to specifically affect the evolution of the photoreaction from **BR**2, where the two possible pathways bifurcate to evolve to **BR**3 or **BR**3B. On the contrary, the energy profile from **BR**2 to **BR**3 is strongly affected by the application of the force. In the absence of external forces (i.e., classical ODPM photorearrangement), the formation of **BR**3 is the only possible pathway, being the preferred path in both thermodynamical and kinetic control, as already discussed in the first section of the results.

When applying a 2 nN external force (see Figure 7), the stability of **BR**3 and **BR**3B is inverted, reaching an energy difference of ca. 23 kcal/mol. Therefore, if the reaction on T_1_ is governed by thermodynamic control, this force is enough to invert the feasibility of each path, making the formation of **BR**3B (classically forbidden) the preferred pathway. It is possible to estimate the force magnitude where the inversion of the thermodynamic stability of **BR**3/**BR**3B is reached by interpolating the energy difference assuming a linear variation: a force magnitude of ca. 0.7 nN makes both biradicals to have similar energies on T_1_, both pathways being equally accessible in the thermodynamic control regime. The energy barriers from **BR**2 to **BR**3 (0.3 kcal/mol) and **BR**3B (6.0 kcal/mol) are not similar under the action of this force magnitude, and the formation of **BR**3B is not expected in the limit of kinetic control. For a 4.2 nN force, the energy difference between **BR**3 and **BR**3B becomes much higher (ca. 66 kcal/mol), making the formation of **BR**3B (the classically forbidden pathway) also the most preferred one in the thermodynamic control regime. Additionally, the energy barrier for each intermediate becomes much closer (i.e., 1.2 kcal/mol), making both biradicals **BR**3 and **BR**3B to have much similar formation velocities, but the velocity of formation of **BR**3 is slightly higher than that of **BR**3B. These results are confirmed by molecular dynamics simulations made from **BR**2. Starting from a sampling following a Boltzmann distribution at 300 K, 20 trajectories for each force (0, 1.5, 3.0, and 4.2 nN) have been computed on T_1_. The formation within 400 fs of **BR**3 and **BR**3B is observed at 4.2 nN, while for other forces, they are not observed as expected (see the Supporting Information for details).

In summary, the formation of **BR**3B is favored in thermodynamic control conditions for magnitude forces >0.7 nN, while for kinetic control conditions, **BR**3 and **BR**3B are formed almost with equal rates for significantly strong forces up to ca. 4.2 nN.

### Ground-State Photoproduct Formation

**Figure 8:**
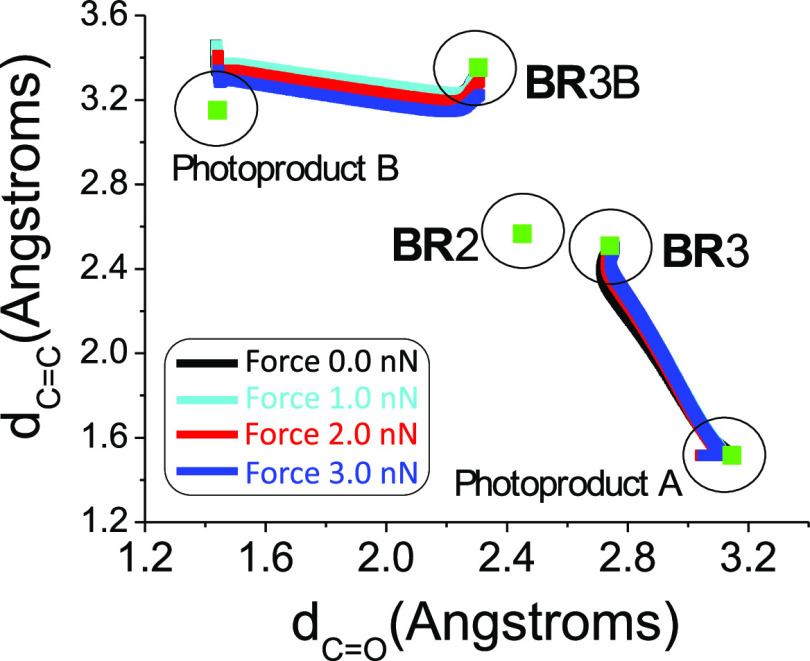
S_0_ minimum energy paths (considering different external forces) from **BR**3 and **BR**3B, after S_0_/T_1_ intersystem crossing. Formation of photoproducts (A from **BR**3 and B from **BR**3B) is always reached for any applied force.

The last step of the reaction is the T_1_/S_0_ intersystem crossing, allowing the population of S_0_ from T_1_. In order to confirm that the corresponding biradicals **BR**3 and **BR**3B lead to the respective photoproducts (i.e., photoproducts A and B, respectively), minimum energy paths in S_0_ have been computed including the external forces. All the computed pathways (considering external forces from 0 to 3 nN) are qualitatively similar (see Figure 8), connecting in all the cases the corresponding biradical with the expected photoproduct.

Additionally, both **BR**3 and **BR**3B in T_1_ are almost degenerated with their respective S_0_. The determined spin–orbit couplings for both species (i.e., in the range of *ca.* 3 cm^–1^ for **BR**3 and *ca.* 10 to 40 cm^–1^ for **BR**3B; see the Supporting Information for details) make the decay to S_0_ an efficient decay path in both cases, regardless of the force magnitude. Nevertheless, it has to be noted that the difference in the spin–orbit couplings for **BR**3 and **BR**3B makes the intersystem rate constant to be ca. 100 times higher for **BR**3B than for **BR**3 due to the quadratic dependence of the rate constant with this magnitude.

A final qualitative prediction of the formed photoproducts can be proposed as a function of the applied force, that is, estimation of the mechanical control on the photochemical reaction. According to the predicted reaction mechanism, and taking into account the effect of the forces on the energetics of each intermediate, the rate of formation of each photoproduct can be estimated.
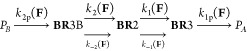


The rate constants are determined as the functions of the applied force magnitude, k_1_(**F**), k_–1_(**F**), k_2_(**F**), and k_–2_(**F**) being the force-dependent rate constants for the respective T_1_ conversions and k_1p_(**F**) and k_2p_(**F**) the T_1_/S_0_ intersystem crossing rates, the values of which are estimated to be in the range of 10^6^–10^8^ s^–1^.

Integrating the corresponding kinetic equations, the population of each photoproduct can be determined as a function of time. A total of 10^3^ kinetic simulations for different temperatures and force magnitudes (ranging from 250 to 450 K and 0 to 4 nN, respectively) were performed. For each simulation, a total of 10^8^ steps of integration were computed (time step of integration of 10 fs), reaching the equilibrium in each case at the end of the simulation (i.e., microsecond timescale). These simulations permit to determine the photoproduct formation rate.

**Figure 9:**
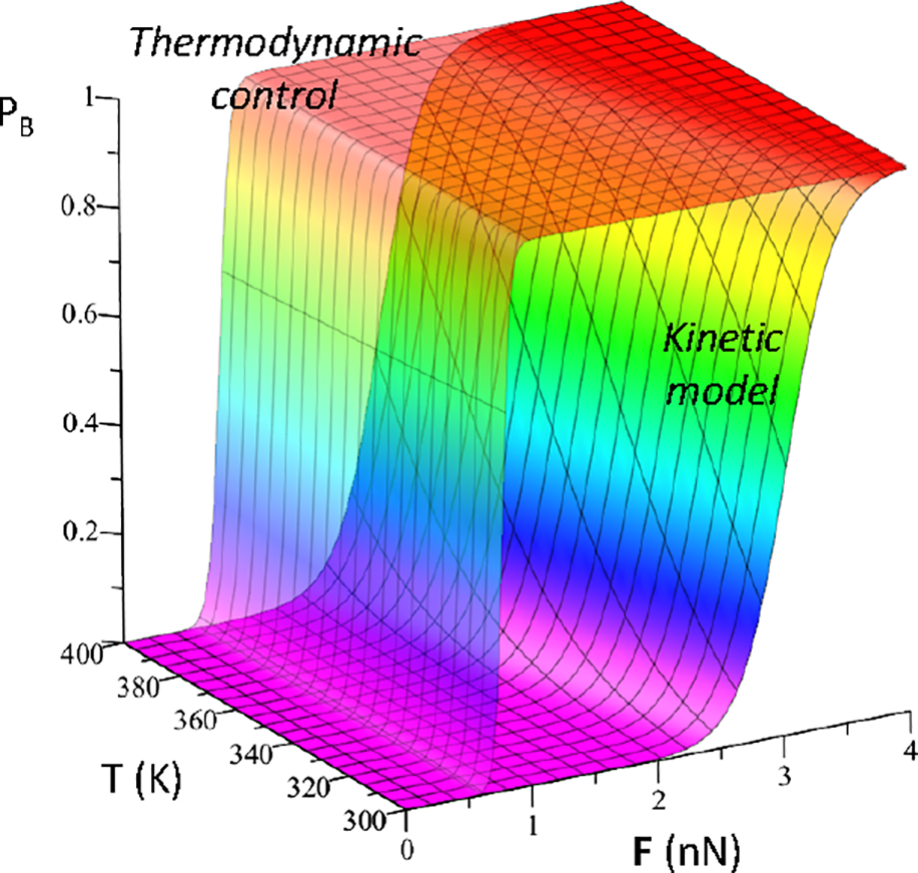
Predictions of photoproduct B (P_B_) abundance for the process studied, considering diverse force magnitudes and temperatures. This abundance ranges from 0 (violet color) to 1 (red color), calculated considering both the thermodynamic control and the kinetic model (see the above reaction mechanism once **BR**2 is formed).

In the limit of thermodynamic control (i.e., assuming that intersystem crossing is the rate-determining step), the relative populations of photoproducts B and A are given by the equilibrium populations of **BR**3 and **BR**3B. Assuming this control, the forbidden photoproduct B is predicted to be the main product even for relatively small forces (*ca.* 0.5 nN) and almost not sensible to temperature in the 300–400 K range (see Figure 9).

On the other hand, it is also possible to estimate specific values for the T_1_/S_0_ intersystem crossing rate constants. Taking into account that k_2p_ is ca. 100 higher than k_1p_ due to the larger spin–orbit coupling values for **BR**3B than for **BR**3, for all the applied forces (see the Supporting Information), the formation of photoproduct B is temperature-dependent, for which ca. 2.5 nN force at 300 K (or ca. 1 nN at 400 K) is necessary to be the predominant photoproduct.

Therefore, the classically forbidden photoproduct B can be the largest one if mechanochemical control is applied. In particular, when applying stretching forces on the specific bond C2–C6, the forbidden photoproduct B is the most abundant in both, and the thermodynamic control and predictions are made on the basis of a kinetic model at relatively soft conditions, that is, 0.5–2 nN and 300–400 K.

## Conclusions

We have shown that the mechanism of the photosensitized ODPM photorearrangement reaction can be altered by means of mechanochemistry. The activation of a classically forbidden pathway is possible in ODPM photorearrangement by including a force pair specifically chosen to favor it. The force pair is selected in view of the mechanism leading to the two possible photoproducts and their respective reaction coordinates. Hence, detailed knowledge of the whole reaction mechanism is mandatory to reach the mechanochemical control of the photoreaction. The external force has been selected, guided by chemical intuition, to affect the mechanism from the bifurcation point (**BR**2). However, caution is required as this strain could affect other properties of the system that can also determine the feasibility of the photoreaction. For instance, ground-sate conformational equilibrium is strongly influenced by the applied force, some of them even vanishing with the force strength. This fact is of crucial importance because if the most stable conformer in the ground state changes, then its photophysical properties could also change, leading to unexpected findings. In this particular case, the conformational equilibrium modification by the force has a direct influence on the triplet excitation energy, which determines the photosensitization feasibility of the system. We demonstrate that, in this case, if an external force is applied, the triplet excitation energy increases. Therefore, it could be experimentally found that the strained ODPM is not photosensitized, not inducing the desired photorearrangement reaction. In that case, the photosensitizer used should be changed, selecting one with a larger triplet energy.

We also demonstrate that the C=O or C=C moieties could be excited after the photosensitization of ODPM, but it is not relevant for the photoproduct formation as the same biradical species will be reached from them. Finally, the overall goal of the forbidden photoproduct activation has been demonstrated to be possible even with relatively small external forces (ca. 0.75 nN), by analyzing the energy profiles computed and simulated in T_1_, considering diverse force strengths. Although this finding could seem trivial, previous works have reported unexpected and counterintuitive behaviors after applying an external force.

Hence, we would like to highlight the importance of performing a complete computational study of a given strained system, covering the study of all the crucial properties that can be affected by the external force, not reducing the study to the analysis of the force effect on the involved intermediates at the relevant electronic state. We hope that these theoretical results for the mechanochemical control of ODPM photorearrangement may encourage experimental photochemists to advance in the development of mechanophotochemistry.

## Supporting Information Available

Computational and methodological details (inclusion of external forces; triplet excitation energy distribution; molecular dynamics simulations with the inclusion of external forces); ground-state conformational equilibrium; spin densities in the triplet state after triplet energy excitation transfer; molecular dynamics simulation of triplet energy transfer between CC and CO moieties with the inclusion of external forces; degeneracy between singlet and triplet states for **BR**3 and **BR**3B; dynamics on T_1_ at different forces from **BR**2, yielding **BR**3 and **BR**3B; spin–orbit couplings in **BR**3 and **BR**3B; and kinetic model for predicting photoproduct formation (PDF)
