# Supplementary material for: Mechanical Activation of Forbidden Photoreactivity in Oxa-di-π-methane Rearrangement
Source: J Org Chem. 2022 Sep 27;87(19):12586–95. doi: 10.1021/acs.joc.2c00720 (PMC9552220; doi:10.1021/acs.joc.2c00720)
Supplement: Supplementary file 1 — jo2c00720_si_001.pdf [file jo2c00720_si_001.pdf]

Supporting Information

Mechanical activation of forbidden photoreactivity in oxa-di-π-methane rearrangement

Alejandro Jodra,[a] *Cristina García-Iriepa*[a,b] *and Luis Manuel Frutos*[a,b,*]

[a] Universidad de Alcalá, Departamento de Química Analítica, Química Física e Ingeniería Química, Grupo de Reactividad y Estructura Molecular (RESMOL), Alcalá de Henares, Madrid, Spain

[b] Universidad de Alcalá, Instituto de Investigación Química "Andrés M. del Río" (IQAR), Alcalá de Henares, Madrid, Spain.

Content

1. Computational and Methodological Details……………………………………………..S2

1.a. DFT Functional Benchmark…………………………………………………………...S2

1.b. Inclusion of External Forces……………………………………………………..…..S2

1.c. Triplet Excitation Energy Distribution…………………………………………..…....S4

1.d. Molecular Dynamics Simulation with inclusion of external forces……………….....S5

2. Ground-state Conformational Equilibrium ………………………….………………..S6

3. Spin densities in the triplet state after triplet energy excitation transfer……………..S9

4. Molecular dynamics simulation of triplet energy transfer between CC and CO moieties with inclusion of external forces. …………………………….…………………...S9

5. Degeneracy between singlet and triplet states for ***BR****3 and* ***BR****3B………...……....S11*

6. Dynamics on T_1 at different forces from ***BR****2 yielding* ***BR****3 and* ***BR****3B. ………..….S11*

7. Spin-orbit couplings in ***BR****3 and* ***BR****3B. ……………………………..………………...S13*

8. Kinetic model for predicting photoproducts formation. …………….………………..S14

9. Cartesian coordinates of the optimized stationary points………….………………....S16

1. Computational and Methodological Details:

1a. DFT Functional Benckmark

To validate the selected level of theory, we have performed a functional benchmark. In particular, the B3LYP and M062X functionals have been considered. The results show similar energy barriers.

Table S1. Energy barriers (in kcal/mol) computed using the B3LYP and M062X functionals, keeping the same 6-31G(d) basis set.

**Energy barrier**	**B3LYP**	**M062X**
BR1→ TS1	13.5	10.7
TS1→BR2	-0.3	-0.9
BR2→TS2	0.1	1.4
BR2→TS2B	13.9	17.6
TS2B→BR3B	-7.9	-8.7
TS2→BR3	-7.4	-7.5

Additionally, the energy profile for T_1 state is qualitatively similar to that found in previous studies performed at CASPT2/CASSCF level of theory.[S1,S2]

1b. Inclusion of External Forces.

From a theoretical and computational point of view, there are two related variants to study mechanochemical phenomena. On the one hand, the COGEF (*Constrained Geometries simulate External Force*) approach for which the external force is simulated by imposing a distance between two nuclei ($q_i = R_0$ where q_i is the coordinate in which the force is applied and R_0 is the value of the corresponding constrained distance). To determine V_{COGEF} potential, it is necessary to use the Lagrange multiplier method. This way, the curve $V_{COGEF}(R_0)$ is obtained for different distance values (R_0).[S3]

The applied external force changes the stationary points on the potential energy surface (PES), and consequently, minima and transition states (TS) will structurally change upon the action of an external force (see Fig. S1). The new minima and TS have to verify the condition: $F_{int} = -F_{ext}$, where F_{int} is the internal forces (determined from Born-Oppenheimer approximation), and F_{ext} is the applied external force.

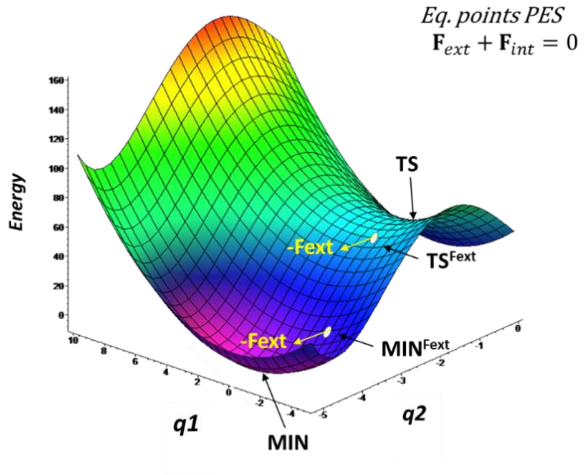

Fig. S1. Representation of the energy as a function of the mechanical *coordinate* (q_2) and another arbitrary coordinate (q_1). The stationary points (minimum -MIN- and TS) are displaced to new structures (indicated by white circles) where the internal force is equal and opposed to the external force (i.e. $F_{int} = -F_{ext}$).

The second approach to include the external force is known as EFEI (External Force is Explicitly Included). It is based in the explicit inclusion of a constant external force, F_0 in the force field and therefore, in the definition of the PES. Therefore, an energy term, due to the mechanical work developed by the external force, is added to the potential energy determined for each configuration, $E_{mech} = -\mathbf{F}_0\mathbf{R}(q)$. In this way, it is possible to obtain the PES at a certain constant force, $V_{EFEI}(\boldsymbol{F}_0)$, permitting the calculation of all possible reaction parameters at this given applied force:

$$V_{EFEI}(\mathbf{F}_0) = V_{BO}(\mathbf{R}_0) - \mathbf{F}_0\mathbf{R}_0 \qquad (S1)$$

where $V_{BO}(\mathbf{R}_0)$ is the Born-Oppenheimer potential. Both potentials (COGEF and EFEI) are related by a Legendre transform at stationary points:[S3]

$$V_{EFEI}(\mathbf{F}_0) = V_{COGEF}(\mathbf{R}_0) - \mathbf{F}_0\mathbf{R}_0 \quad (S2)$$

Both approaches (COGEF and EFEI) are used in this work. The first one is applied to determine minima and transition states structures, and the second one for describing the dynamics of the studied system.

Regarding the applied force, as it is defined in the main text, an extension force has been applied indirectly to a couple of specific atoms through methyl groups directly linked to these atoms. In this way, we obtain a differential effect on both reaction coordinates. The magnitude of the force applied to atoms “A” and “B” is defined as: $|\mathbf{F}| = \mathbf{F}_A = -\mathbf{F}_B$.[S4]

1c. Triplet Excitation Energy Distribution

The T_1-S_0 spectrum has been simulated by considering the contribution of the 8 conformers in the ground state. Therefore, for each conformer, the triplet excitation energy distribution has been determined at a given temperature, and then, the final distribution has been obtained by summing up all the contributions weighted by the abundance of each conformer at the selected temperature.

In order to simulate the triplet excitation energy distribution of each individual conformer, sampling of the phase space was carried out at 300K following a Boltzmann distribution. In this sampling, the external forces have been explicitly included, affecting only the equilibrium geometries, so for every applied force a new minimum on S_0 has been determined. The potential energy surface (second order approach) necessary to compute the potential energy in the distribution is not affected by the linear term in the

energy included by the force, not affecting the force constants necessary to perform the sampling.

For each sampling structure, the T_1 energy is computed, being possible to construct from this data a normalized gaussian distribution function for the triplet excitation energy for each conformer. Subsequently, by summing up the individual gaussian distribution functions of each isomer weighted by their equilibrium population at 300K, the final triplet excitation energy distribution is obtained for different forces (from 0nN to 2nN). Thus, the triplet energy distribution of the system at a given force in thermal equilibrium would be determined by Eq. S3.

$$g_t = \sum_{i=1}^{N} p_i g_i \qquad (S3)$$

where p_i is the population of the conformer "i" at 300K, and g_i is the normalized Gaussian function corresponding to the spectrum of this conformer:

$$g(x) = \frac{1}{\sqrt{2\pi}\omega_i} e^{-\frac{(v-v_0^i)^2}{2\omega_i}} \qquad (S4)$$

where v_0^i is the vertical triplet excitation energy of conformer "i", and ω_i is the full width at half maximum for the corresponding "i" conformer. The distributions fulfill:

$$\int_0^\infty \frac{1}{\sqrt{2\pi}\omega_i} e^{-\frac{(v-v_0^i)^2}{2\omega_i}} dx = 1 \qquad (S5)$$

1d. Molecular Dynamics Simulation with inclusion of external forces.

In order to describe the dynamical behavior of the system, classical dynamics have been performed using the Velocity-Verlet algorithm.[S5] A time-step of 0.4fs has been employed for propagating the nuclei dynamics. When external forces are applied, the explicit force pair is included within the *DFT* force field. The force pair applied to atoms "i" and "j" is given by:

$$F_{ext}^{ij} = |F_{ext}| \boldsymbol{u}_{ij} \quad (S6)$$

where $\boldsymbol{u}_{ij} = \frac{\boldsymbol{R}_i - \boldsymbol{R}_j}{|\boldsymbol{R}_i - \boldsymbol{R}_j|}$ is the unitary vector pointing from "j" atom to "i" atom, being $|F_{ext}|$ the magnitude of the applied force. The equations of motion are integrated with the new force field including the external forces.

In order to determine the initial sampling for the dynamics, a classical distribution (Boltzmann distribution) at 300K has been performed. The same approach has been employed when external forces are included. It has to be noted that the application of the external forces only changes the equilibrium structure, but does not actually affect the geometry distribution obtained, since the external forces do not alter the force constants (i.e. second derivatives of the energy).

2. Ground-state conformational equilibrium.

As discussed in the main text, 8 possible isomers could be populated in the ground state (see Fig. S2).

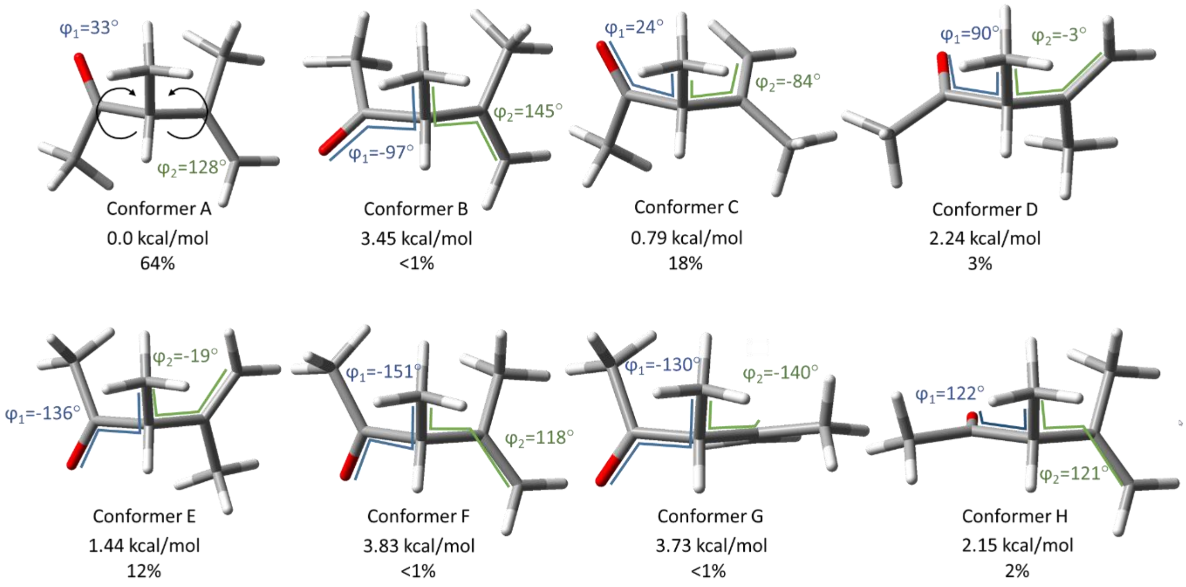

Fig. S2. *Structure of the 8 possible conformers including their structural information, relative energies (in kcal/mol) and the population at 300K (percentage).*

The equilibrium populations in the absence of external forces are indicated in Fig. S2. These populations show that at 0nN force, the photochemistry is mainly governed by the isomer A. Nevertheless, the inclusion of an external force pair (as described in the main text) changes these equilibrium populations in a complex way.

To determine the population of each conformer for different external force magnitudes, we performed a sequence of restricted optimizations, lengthening the C1-C6 distance to each isomer, and determining the stationary points for each considered force (COGEF approximation).

The inclusion of the external force pair makes the topology of the potential energy surface to be affected, making some stationary points to be displaced in terms of coordinates, changing the relative energy among them and eventually even to disappear (*i.e.* being no longer minima on the potential energy surface). This behavior is at the origin of the changes in the population of each isomer as a function of the applied force. For the studied ODMP system, the potential energy surfaces become largely affect by the inclusion of external forces (see Fig. 4 in the main text).

Initially (F=0nN) all the 8 conformers are present with different stability, and therefore different equilibrium populations. Conformer A has the largest population (ca. 64%). For small force magnitude (0.5nN), most of the isomers do not correspond to any minimum on the potential energy surface as they are no longer stable, and therefore, their population is vanishing. In fact, only isomers “C”, “D” and “E” are present. Finally, for larger force magnitudes (e.g. up to 1.5nN), only isomers “D” and “E” are stable (see Fig. S3). A minimum on the potential energy surface (associated with a stable isomer) disappears when the applied force exceeds a certain limit (F_{max}). The results of the F_{max} for the different isomers are summarized in Table S1.

***Table S2**. Values of the maximum force magnitude that can be applied for each conformer after losing its stationary point character and therefore disappearing. For larger forces each conformer transforms into another one (indicated in the table) by minimum energy path.*

Conformer	***A***	***B***	***C***	***D***	***E***	***F***	***G***	***H***
F_{max} *(nN)*	0.74	0.52	1.31	5.67	5.63	0.42	0.25	0.74
F > F_{max}	C	E	D	--	--	E	E	D

These results can be explained in terms of coupling between mechanical coordinates (*i.e.* the distance between methyl groups where the force pair is applied) and relevant torsions (*i.e.* φ_1 and φ_2). More specifically, the mechanical coordinate is strongly coupled with φ_2, in such a way that applying stretching forces, makes the mechanical energy to largely change as the torsion approaches to zero, therefore, stabilizing conformers with φ_2 dihedrals close to this value. In this case, conformers D and E are the most favored (see Fig. S3) and therefore they tend to persist in the equilibrium as the forces grow up.

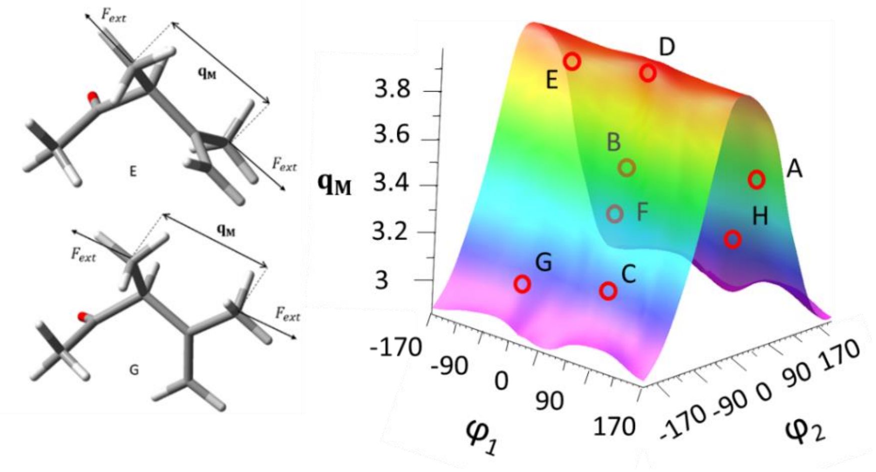

***Fig. S3**. (left) Mechanical coordinate (**q_M**) is defined as the distance between methyl carbon atoms where the force pair is applied. (right) Coupling between the mechanical coordinate (**q_M**) and the dihedral angles φ_1 and φ_2 obtained from relaxed scan on the ground state. The surface shows that D and E are the most stabilized isomers due to the mechanical effect of the applied force.*

3. Spin densities in the triplet state after triplet energy excitation transfer.

The two triplet excited states of ODMP correspond to the local excitation in the C=C and C=O moieties.[S2] In order to follow the type of triplet excitation (CC* or CO*), the spin density has been analysed (see Fig. S4).

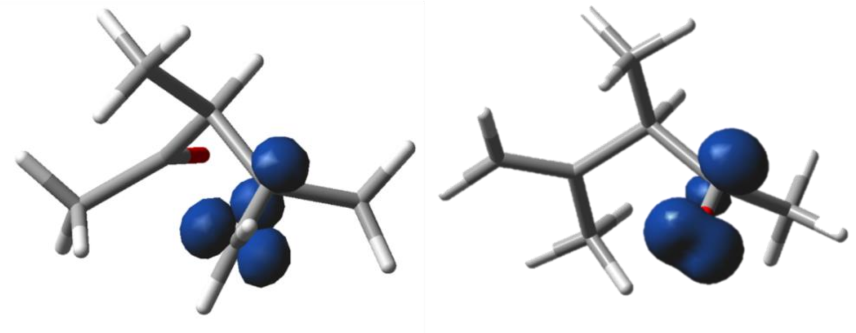

Fig. S4. *Representation of the spin density for* ***BR****1 localized on C=C moiety (left) and C=O moiety (right)*.

4. Molecular dynamics simulation of triplet energy transfer between CC and CO moieties with inclusion of external forces.

In order to look for potential triplet energy migration, during molecular dynamics simulations the wavefunction has been determined on each point of the dynamics from the scratch, in order to correctly identify the lowest lying triplet state. In this way, when the two triplet states (corresponding to C=C and C=O triplet excitations) cross, the system remains in the lowest state, and therefore, a hop event is detected. Hoping between states is identified by analysing the spin density on the different moieties. To identify where the spin density is located to the greatest extent, the spin density of each chromophore (C=C and C=O respectively) is summed up. The difference between both spin densities (*i.e.* spin density of C=C minus that of C=O) provides an easy parameter to interpret, in such a way that for positive values the diradical character corresponds to the C-C bond, while for negative values the diradical can be assigned to the C-O bond.

Figure S5 shows the simulations made for the most abundant conformer at different applied strength forces.

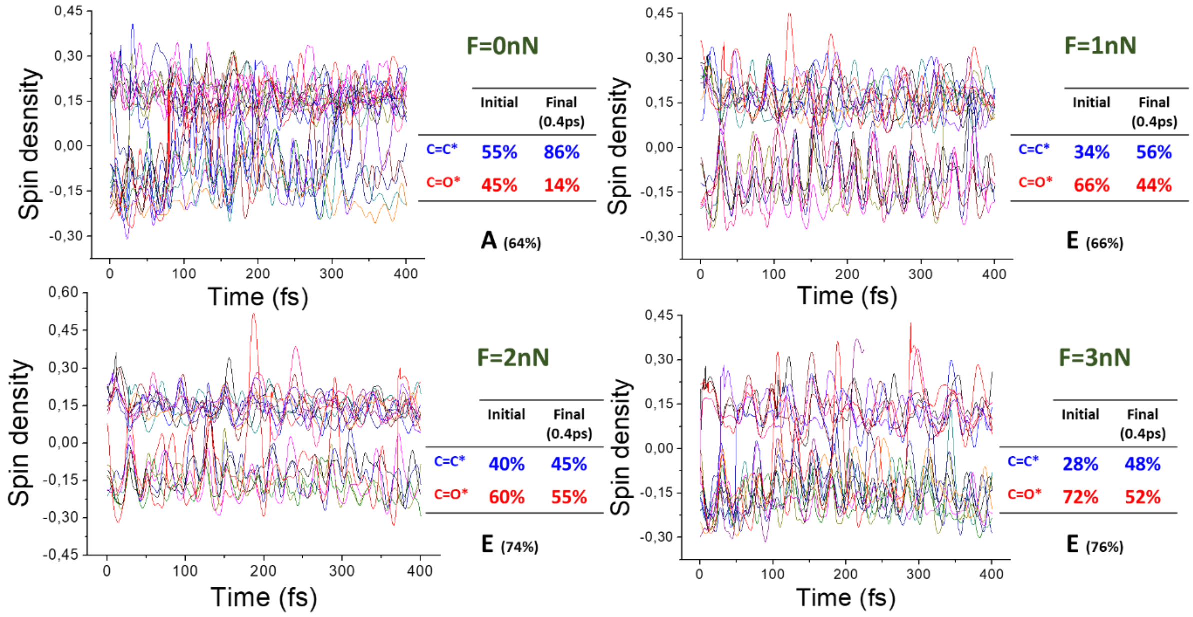

Fig. S5. *Evolution of simulated trajectories after vertical excitation to T_1 corresponding to all the conformers at force 0nN. The spin density is the difference between the atomic spin densities of CC moiety (i.e. the sum of the atomic spin densities for each carbon atom) and the CO moiety (i.e. the sum of the atomic spin densities for the carbon and the oxygen atom). Positive values indicate localization of the triplet excitation on the CC moiety, while negative values on the CO.*

5. Degeneracy between singlet and triplet states for BR3 and BR3B.

The singlet-triplet energy gap in **BR**3 and **BR**3B has been studied for different force magnitudes. For both **BR**3 and **BR**3B the stable conformation changes as indicated in Figure S6. In both cases, the energy degeneracy is basically fulfilled for all the force magnitudes considered.

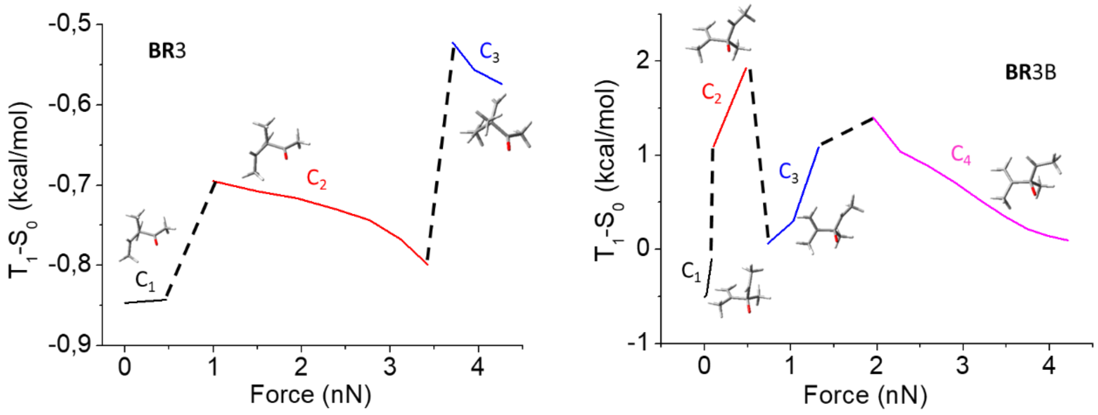

***Fig. S6**. T_1-S_0 energy difference for **BR**3 and **BR**3B at different force magnitudes. The stable conformation for each conformer is indicated.*

*6. Dynamics on T_1 at different forces from **BR**2 yielding **BR**3 and **BR**3**B**.*

A series of 20 molecular dynamics trajectories has been simulated starting from **BR**2 (sampling obtained at 300K from a Boltzmann distribution and explicit inclusion of external forces as explained above) for each external force magnitude considered (i.e. 0nN, 1.5nN, 3.0nN and 4.2nN). The trajectories can be splited into two sets, the first one corresponding to q_1 (C_2-C_3 distance) and the second one to q_2 (C_2-C_5 distance). The first path is related to C_2-C_3 bond-breaking, and therefore with the formation of the classical photoproduct, while the second is related to C_2-C_5 bond-breaking, leading to classically forbidden photoproducts. Large force magnitudes permit to identify the

formation of the classically forbidden product in the hundreds of femtosecond time-scale (specifically 400fs).

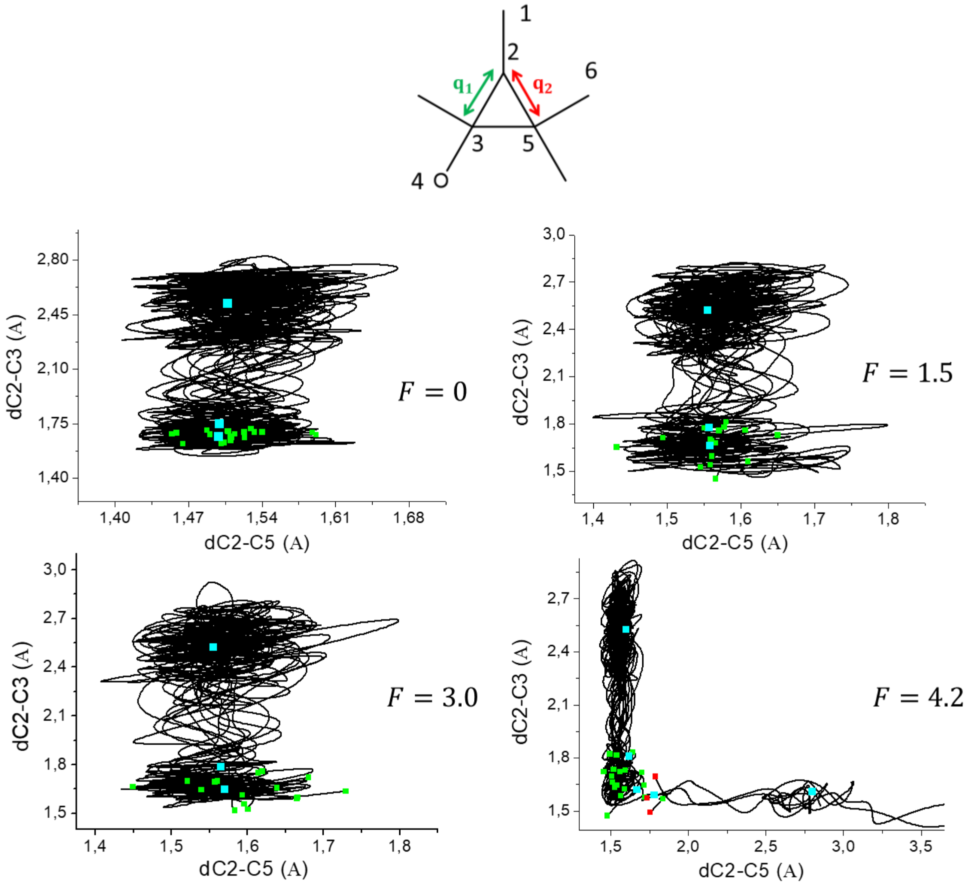

*Fig. S7. (up) Coordinates q_1 (C_2-C_3 distance) and q_2 (C_2-C_5 distance). (down) MD trajectories from **BR**2 for different external forces (indicated in nN units) as a function of coordinates q_1 and q_2. It is observed that only for very large forces (up to 4.2nN) the second path is reached in a very short time-scale (i.e. 400fs). Starting points of each trajectory are indicated in green (when evolving to **BR**3) and red (when evolving to **BR**3B). The two minima (**BR**2 and **BR**3 or **BR**3B) and the transition state (**TS**2 or **TS**2B) are indicated with light blue dots.*

*7. Spin-orbit couplings in **BR**3 and **BR**3B*

Determination of spin-orbit couplings (SOC) for different conformers as a function of the applied force magnitude (most stable conformers at a given force are displayed in Figure S8). The SOC is *ca.* 10 times larger in **BR**3B than in **BR**3, and therefore the corresponding intersystem crossing (ISC) rate constant is *ca.* 100 higher for **BR**3B than in **BR**3. SOC values have been determined with ORCA using B3LYP functional and the 6-31G(d) basis set.[S6]

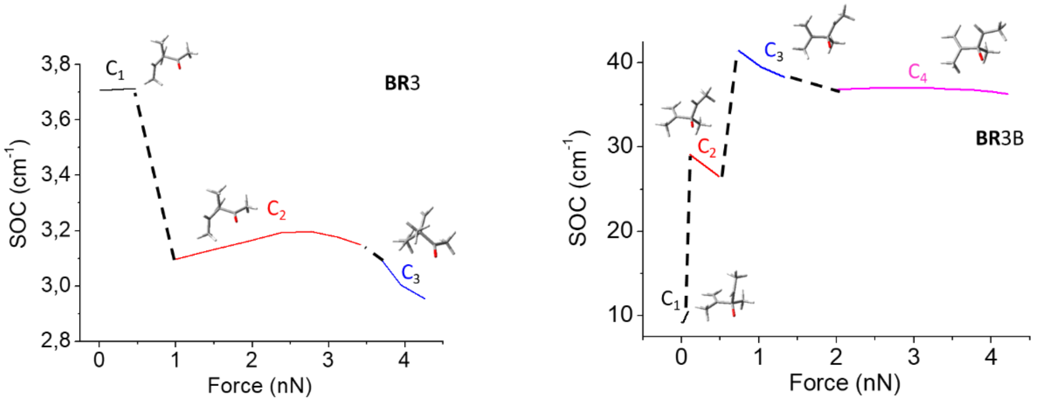

***Fig. S8**. SOC values for **BR**3 and **BR**3B as a function of the applied force.*

8. Kinetic model for predicting photoproducts formation.

Once the ODPM is excited to triplet state, the system evolves to yield **BR**2 independently on the applied forces. From this point the reaction path bifurcates, and two different pathways are feasible, one corresponding to the formation of photoproduct A (*i.e.* **BR**2→[**TS**2]$^{\neq}$→**BR**3→Photoproduct **A**) and the second to the formation of photoproduct B (*i.e.* **BR**2→[**TS**2B]$^{\neq}$→**BR**3B→Photoproduct **B**). Therefore, the relative photoproduct formation P_A/P_B depends on the relative rates for both reaction pathways. The two pathways are summarized in the following:

$$P_B \xleftarrow{k_{2p}(\mathbf{F})} \mathbf{BR}3\mathrm{B} \underset{k_{-2}(\mathbf{F})}{\overset{k_2(\mathbf{F})}{\leftrightarrows}} \mathbf{BR}2 \underset{k_{-1}(\mathbf{F})}{\overset{k_1(\mathbf{F})}{\rightleftarrows}} \mathbf{BR}3 \xrightarrow{k_{1p}(\mathbf{F})} P_A$$

where k_1, k_{-1}, k_2 and k_{-2} are the rate constants for thermally activated steps corresponding to the equilibrium of **BR**2 with **BR**3 and **BR**3B. These rate constants can be estimated from computed energy barriers, and in fact, the energy barriers depend on the applied force magnitude. From the computed reaction energy profiles determined at 0nN, 2.0nN and 4.2nN it is possible to estimate the rate constants as a function of the force magnitude **F**:

$$k_1(\mathbf{F}) = \frac{k_B T}{h} e^{-\frac{0.1 + 0.03939\cdot\mathbf{F} + 0.0303\cdot\mathbf{F}^2}{RT}} \qquad k_{-1}(\mathbf{F}) = \frac{k_B T}{h} e^{-\frac{7.4 + 0.2026\cdot\mathbf{F} - 0.2013\cdot\mathbf{F}^2}{RT}}$$

$$k_2(\mathbf{F}) = \frac{k_B T}{h} e^{-\frac{13.9 - 4.96515\cdot\mathbf{F} + 0.50758\cdot\mathbf{F}^2}{RT}} \qquad k_{-2}(\mathbf{F}) = \frac{k_B T}{h} e^{-\frac{7.9 + 12.56645\cdot\mathbf{F} + 0.64177\cdot\mathbf{F}^2}{RT}}$$

where k_B is the Boltzmann's constant, h the Planck's constant, and T is the absolute temperature. The units of the ideal gas constant in the above expressions are $R = 1.989 \cdot 10^{-3}\, kcal \cdot mol^{-1} \cdot K^{-1}$, therefore, the activation energies are expressed in kcal·mol^{-1} and **F** (*i.e.* the force magnitude) in nanonewtons (nN). The ratio of the intersystem rate constants $k_{1p}(\mathbf{F})$ and $k_{2p}(\mathbf{F})$ can be estimated by assuming that spin orbit coupling values for each reacting species (*i.e.* **BR**3 and **BR**3B respectively) is the

determining factor distinguishing between both rate constants. Since SOC is *ca.* 10 times for **BR**3B than for **BR**3 (see Figure S8), the ratio between ISC rate constants is:

$$\frac{k_{2p}(\mathbf{F})}{k_{1p}(\mathbf{F})} \sim 100$$

According to these values, the rate equations for the different species are the following (taking **BR**2 as the starting point):

$$\frac{d[\mathbf{BR}2]}{dt} = k_{-1}(\mathbf{F})[\mathbf{BR}3] + k_{-2}(\mathbf{F})[\mathbf{BR}3B] - \{k_1(\mathbf{F}) - k_2(\mathbf{F})\}[\mathbf{BR}2]$$

$$\frac{d[\mathbf{BR}3]}{dt} = k_1(\mathbf{F})[\mathbf{BR}2] - \{k_{-1}(\mathbf{F}) + k_{1p}(\mathbf{F})\}[\mathbf{BR}3]$$

$$\frac{d[\mathbf{BR}3B]}{dt} = k_2(\mathbf{F})[\mathbf{BR}2] - \{k_{-2}(\mathbf{F}) + k_{2p}(\mathbf{F})\}[\mathbf{BR}3B]$$

$$\frac{d[P_A]}{dt} = k_{1p}(\mathbf{F})[\mathbf{BR}3]$$

$$\frac{d[P_B]}{dt} = k_{2p}(\mathbf{F})[\mathbf{BR}3B]$$

Numerically integrating this set of differential equations, the ratio of the photoproducts can be determined. This ratio for a wide range of force magnitudes and temperatures (ranging from 250K to 450K temperature and 0nN to 4nN force magnitudes) were performed in order to obtain the results summarized in Figure 9 of the main text. For each simulation, a total of 10^8 steps of integration were computed (time step of integration of 10fs), reaching the equilibrium in each case at the end of the simulation (*i.e.* microseconds time-scale).

9. Cartesian coordinates of the optimized stationary points.

**S_0 minimum**				**T_1 BR1**			
6	1.830039	0.267383	1.182842	6	-2.575742	-0.320837	-0.216069
6	1.204515	-0.402842	-0.028641	6	-1.104518	-0.501249	0.136569
8	1.605393	-1.476846	-0.436513	8	-0.749546	-1.382264	0.896078
6	0.010814	0.300007	-0.712238	6	-0.095574	0.481000	-0.507974
6	-1.258745	-0.062820	0.059833	6	1.330550	0.114089	-0.168857
6	-1.910249	0.802352	0.845278	6	1.891358	0.474061	1.133202
1	1.061455	0.618866	1.879406	6	1.991098	-0.981307	-0.961408
1	2.494301	-0.440911	1.681836	6	-0.452416	1.939533	-0.139005
1	-0.058233	-0.180020	-1.696601	1	-2.717228	-0.034016	-1.263875
6	0.241523	1.804441	-0.912707	1	-3.113303	-1.247694	-0.003303
6	-1.733169	-1.484818	-0.123007	1	-3.008043	0.476349	0.401830
1	-2.806274	0.503465	1.383303	1	-0.239558	0.371229	-1.595649
1	-1.596189	1.833383	0.975312	1	2.576296	1.312017	1.258599
1	-0.613316	2.251633	-1.430131	1	1.696726	-0.137949	2.014890
1	2.415102	1.142045	0.872585	1	3.083988	-0.896688	-0.933794
1	-2.584000	-1.711042	0.526574	1	1.735947	-1.973019	-0.550385
1	-2.041441	-1.657770	-1.163260	1	1.671481	-0.973509	-2.011919
1	1.135453	1.979088	-1.521865	1	0.245773	2.627774	-0.625788
1	0.374221	2.338546	0.033214	1	-0.379206	2.097691	0.942227
1	-0.932593	-2.204736	0.083573	1	-1.465041	2.204195	-0.460197
**T_1 TS1**				**T_1 BR2**			
6	-1.587577	0.803991	0.992450	6	1.498187	-0.929724	1.023755
6	-0.498722	0.788495	-0.072886	6	0.374785	-0.831088	-0.000106
8	-0.205615	1.882213	-0.662072	8	0.103465	-1.864859	-0.707783
6	-0.204934	-0.553963	-0.877335	6	0.241429	0.575934	-0.883632
6	0.814706	-0.265097	0.158803	6	-0.772305	0.272035	0.178094
6	0.837071	-1.005139	1.432183	6	-0.786942	1.064839	1.416168
1	-1.809905	-0.173926	1.424384	1	2.315373	-1.511630	0.586827
1	-2.505890	1.196847	0.542003	1	1.894638	0.032464	1.355525
1	0.098335	-0.291919	-1.889536	1	-0.068807	0.311852	-1.891723
6	-1.164931	-1.722319	-0.804763	6	1.304355	1.639558	-0.806990
6	2.151888	0.298019	-0.296810	6	-2.147496	-0.164240	-0.312771
1	0.282669	-0.694044	2.310053	1	0.095776	1.243839	2.016745
1	1.529286	-1.836879	1.542792	1	-1.703402	1.567593	1.706918
1	-0.707821	-2.599795	-1.279953	1	2.211863	1.332761	-1.339347

1	-1.300743	1.485561	1.801089	1	1.136294	-1.467317	1.908377
1	2.026207	0.940739	-1.170182	1	-2.676016	-0.720258	0.468704
1	2.827769	-0.528035	-0.556862	1	-2.748126	0.715932	-0.573605
1	-1.409469	-2.001366	0.223491	1	0.932395	2.552556	-1.291869
1	-2.096037	-1.502076	-1.338457	1	1.573118	1.905998	0.217469
1	2.625519	0.883269	0.497903	1	-2.062905	-0.808799	-1.188868
**T_1 TS2**				**T_1 TS2B**			
6	1.462180	-1.032806	0.987454	6	-1.166151	0.959870	1.250686
6	0.316534	-0.879887	-0.003688	6	-0.202349	0.718272	0.084384
8	0.019903	-1.853997	-0.759684	8	0.137278	1.828712	-0.574538
6	0.251065	0.634389	-0.879916	6	-0.654580	-0.323472	-0.976684
6	-0.743261	0.285788	0.187031	6	0.900108	-0.328297	0.247725
6	-0.725429	1.042518	1.450027	6	0.867601	-1.291123	1.234738
1	2.240432	-1.648228	0.527032	1	-1.974769	1.610046	0.904566
1	1.907024	-0.091916	1.319295	1	-1.602672	0.044799	1.657382
1	-0.062021	0.388775	-1.891282	1	-0.259217	-0.152069	-1.969123
6	1.361064	1.638518	-0.768389	6	-1.815417	-1.236926	-0.763847
6	-2.142589	-0.067264	-0.309372	6	2.159726	-0.105157	-0.560607
1	0.176156	1.212796	2.024841	1	0.031406	-1.417552	1.912049
1	-1.639084	1.513422	1.796354	1	1.702006	-1.975507	1.353486
1	2.250251	1.313891	-1.321303	1	-2.766656	-0.694181	-0.658622
1	1.095742	-1.560002	1.877129	1	-0.632172	1.472255	2.057419
1	-2.690445	-0.623800	0.458399	1	2.768359	0.674303	-0.084584
1	-2.702509	0.847617	-0.537620	1	2.754673	-1.022223	-0.613777
1	1.032369	2.589205	-1.213009	1	-1.914040	-1.912978	-1.619941
1	1.647988	1.850659	0.263830	1	-1.691251	-1.856301	0.133950
1	-2.092499	-0.687981	-1.205081	1	1.952489	0.240713	-1.574875
**T_1 BR3**				**T_1 BR3B**			
6	-2.239111	-0.557562	0.465033	6	-0.954456	-0.757314	1.455456
6	-0.842038	-0.678221	-0.127944	6	-0.204673	-0.596263	0.118105
8	-0.468057	-1.717533	-0.629246	8	0.156119	-1.824212	-0.387791
6	0.119024	0.585761	-0.037548	6	-1.105891	0.014887	-0.971937
6	1.504232	0.179603	-0.472718	6	1.041807	0.330560	0.184343
6	2.415944	-0.673013	0.344741	6	1.185372	1.260425	1.135182
6	-0.442368	1.640543	-1.029631	1	-1.360680	0.185299	1.832050
6	0.068918	1.108842	1.363910	1	-1.781478	-1.457975	1.309781
1	-2.666176	0.442858	0.353565	1	-0.869705	-0.259223	-1.994409
1	-2.891041	-1.300306	-0.001283	6	-1.997507	1.175422	-0.683243

1	-2.184688	-0.771978	1.539584	6	2.071071	0.123992	-0.898813
1	1.699599	0.233998	-1.541611	1	2.057466	1.909826	1.147123
1	3.431434	-0.667239	-0.066620	1	0.468362	1.409680	1.935518
1	2.481385	-0.349551	1.391318	1	-2.467875	1.531695	-1.605027
1	2.074121	-1.721859	0.353043	1	-0.277440	-1.167288	2.210773
1	0.220576	2.511485	-1.046710	1	-2.800115	0.920617	0.023457
1	-0.496908	1.230236	-2.044339	1	-1.443657	2.019067	-0.241235
1	-1.441985	1.983079	-0.743273	1	1.621590	0.165716	-1.897689
1	-0.723596	1.782990	1.673554	1	2.538909	-0.862754	-0.811788
1	0.734121	0.730840	2.131687	1	2.851341	0.888785	-0.840782

Force 2.0 nN

**S_0 minimum**				**T_1 BR1**			
6	1.779893	0.132253	1.149035	6	-1.809632	0.159318	1.101882
6	1.203777	-0.368351	-0.165946	6	-1.162935	-0.448604	-0.133673
8	1.669864	-1.338372	-0.733455	8	-1.495864	-1.545539	-0.545833
6	-0.012175	0.382039	-0.747679	6	-0.027018	0.341066	-0.819679
6	-1.272665	-0.061033	0.045943	6	1.253676	-0.018578	-0.023111
6	-2.015166	0.832859	0.709451	6	1.874294	0.970776	0.854168
1	0.986460	0.400788	1.854377	6	1.824315	-1.460806	0.022439
1	2.425322	-0.637148	1.577759	6	-0.410444	1.862661	-1.017928
1	-0.114741	-0.023384	-1.762817	1	-2.516926	0.944224	0.804909
6	0.303884	1.924976	-0.881702	1	-2.357430	-0.616304	1.641394
6	-1.667656	-1.561978	0.068668	1	-1.069103	0.625967	1.760603
1	-2.901438	0.533708	1.263809	1	0.059534	-0.119636	-1.812936
1	-1.782273	1.893559	0.718427	1	2.697783	1.601554	0.517168
1	-0.519544	2.436407	-1.389579	1	1.602463	1.069758	1.907274
1	2.381462	1.033399	0.974851	1	1.836716	-1.838390	1.053664
1	-2.568762	-1.700842	0.670657	1	1.228649	-2.144863	-0.589808
1	-1.860301	-1.929171	-0.947081	1	2.861733	-1.470694	-0.338699
1	1.211724	2.051659	-1.479273	1	0.365624	2.375864	-1.594146
1	0.455470	2.401007	0.091639	1	-0.524352	2.384811	-0.063733
1	-0.864528	-2.182968	0.481708	1	-1.352175	1.924240	-1.572194
**T_1 TS1**				**T_1 BR2**			
6	-1.545145	0.778524	0.977821	6	1.449808	-0.911228	1.010291
6	-0.487724	0.733824	-0.118953	6	0.352090	-0.773386	-0.036522

8	-0.204500	1.815960	-0.733294	8	0.116248	-1.782064	-0.798863
6	-0.217199	-0.625319	-0.887916	6	0.262008	0.647885	-0.888532
6	0.858529	-0.296597	0.122220	6	-0.813329	0.297972	0.152896
6	0.883975	-1.023076	1.404939	6	-0.837701	1.098815	1.384347
1	-1.735013	-0.182695	1.460002	1	2.324341	-1.381688	0.549747
1	-2.484744	1.133151	0.539061	1	1.757381	0.031412	1.467333
1	0.060130	-0.383971	-1.913362	1	-0.029242	0.412817	-1.910058
6	-1.301724	-1.752289	-0.816603	6	1.455036	1.640351	-0.809732
6	2.225510	0.388717	-0.236375	6	-2.228920	-0.245153	-0.251130
1	0.369030	-0.678825	2.295166	1	0.059754	1.469924	1.863478
1	1.563541	-1.865711	1.514559	1	-1.789774	1.446423	1.770514
1	-0.944357	-2.637073	-1.355451	1	2.321295	1.203011	-1.315852
1	-1.247613	1.502675	1.744257	1	1.095190	-1.571019	1.810696
1	2.134018	0.983878	-1.146455	1	-2.617307	-0.841881	0.579001
1	2.995646	-0.380013	-0.373583	1	-2.914179	0.588200	-0.441517
1	-1.526829	-2.049267	0.210406	1	1.184867	2.566740	-1.330934
1	-2.217428	-1.397883	-1.298891	1	1.732644	1.897074	0.214453
1	2.523704	1.040567	0.588747	1	-2.164530	-0.876757	-1.137728
**T_1 TS2**				**T_1 TS2B**			
6	1.417473	-1.009185	0.972571	6	-1.103519	0.940031	1.252791
6	0.288737	-0.834280	-0.033284	6	-0.207060	0.677915	0.040601
8	0.028323	-1.778662	-0.836047	8	0.045696	1.742239	-0.702534
6	0.267239	0.719089	-0.884394	6	-0.641139	-0.487126	-0.931641
6	-0.781426	0.313858	0.161484	6	0.901152	-0.378525	0.136719
6	-0.773885	1.077468	1.420665	6	0.913471	-1.343421	1.161028
1	2.267671	-1.489971	0.479196	1	-1.975413	1.516599	0.929644
1	1.752110	-0.082146	1.441566	1	-1.445872	0.032292	1.754032
1	-0.023968	0.494654	-1.907909	1	-0.326790	-0.295358	-1.952531
6	1.511903	1.633579	-0.772742	6	-1.987233	-1.225670	-0.746550
6	-2.228347	-0.143966	-0.251947	6	2.274387	-0.010758	-0.502776
1	0.136781	1.437381	1.883817	1	0.039245	-1.583742	1.754359
1	-1.714869	1.380854	1.865945	1	1.803793	-1.939425	1.331778
1	2.361845	1.157506	-1.272558	1	-2.822722	-0.525529	-0.873673
1	1.069604	-1.678278	1.769101	1	-0.547358	1.539741	1.980999
1	-2.639162	-0.747427	0.562305	1	2.734915	0.764119	0.119490
1	-2.871835	0.727836	-0.411362	1	2.928258	-0.886358	-0.536243
1	1.305982	2.581913	-1.285741	1	-2.073089	-2.009224	-1.506207
1	1.786793	1.862179	0.258886	1	-2.077459	-1.692803	0.239146

1 -2.198100 -0.750705 -1.157503	1 2.161107 0.398188 -1.508647
**T_1 BR3**	**T_1 BR3B**
6 -2.182304 -0.572095 0.535395	6 -0.513943 -0.693356 1.303449
6 -0.833973 -0.633653 -0.167258	6 -0.216442 -0.135451 -0.144505
8 -0.498426 -1.624499 -0.782015	8 -0.251292 -1.292933 -0.901012
6 0.124146 0.625161 -0.071292	6 -1.335113 0.837802 -0.576922
6 1.546113 0.166132 -0.411646	6 1.236647 0.472346 -0.120338
6 2.392060 -0.833625 0.391322	6 1.386156 1.794263 0.013681
6 -0.518230 1.682755 -1.098551	1 -0.545196 0.158054 1.988241
6 0.065676 1.204840 1.309581	1 -1.472951 -1.216557 1.316489
1 -2.669804 0.401591 0.428131	1 -1.027546 1.552730 -1.337853
1 -2.827469 -1.359856 0.140005	6 -2.851195 0.689814 -0.387549
1 -2.032978 -0.742264 1.608670	6 2.484622 -0.441784 -0.198422
1 1.880191 0.354147 -1.430532	1 2.371892 2.250873 0.063901
1 3.458076 -0.621245 0.253071	1 0.538070 2.468848 0.086788
1 2.169620 -0.804064 1.464055	1 -3.339569 1.642289 -0.614164
1 2.203137 -1.859892 0.042632	1 0.272924 -1.385047 1.611043
1 0.117508 2.571952 -1.142449	1 -3.267669 -0.071387 -1.065725
1 -0.594933 1.248183 -2.100890	1 -3.122892 0.395940 0.633249
1 -1.512931 1.980915 -0.758522	1 2.499262 -1.005200 -1.137344
1 -0.827136 1.709469 1.666517	1 2.494694 -1.178442 0.614267
1 0.895042 1.099018 1.999285	1 3.388101 0.168138 -0.128200

Force 4.2 nN

**S_0 minimum**	**T_1 BR1**
6 1.776159 0.132212 1.135940	6 -1.802175 0.128946 1.079673
6 1.204020 -0.327913 -0.195962	6 -1.162203 -0.410580 -0.191302
8 1.669177 -1.285185 -0.786909	8 -1.509341 -1.477107 -0.667918
6 -0.006960 0.438455 -0.753740	6 -0.025515 0.408418 -0.825025
6 -1.289769 -0.114949 0.040476	6 1.276146 -0.065691 -0.009126
6 -2.059811 0.771990 0.681910	6 1.958383 0.929728 0.811294
1 0.979581 0.383778 1.844055	6 1.865720 -1.577459 0.124817
1 2.416581 -0.651215 1.546619	6 -0.514461 1.987465 -1.024411
1 -0.106312 0.075310 -1.783879	1 -2.532194 0.908364 0.825914
6 0.398677 2.050556 -0.895878	1 -2.326231 -0.680016 1.593368
6 -1.726996 -1.681675 0.134235	1 -1.061706 0.583561 1.746517

1	-2.946508	0.470673	1.235451	1	0.076174	0.001240	-1.838938
1	-1.846022	1.837791	0.670338	1	2.795719	1.514874	0.428083
1	-0.394089	2.584831	-1.425358	1	1.697549	1.103648	1.857372
1	2.382010	1.035691	0.990931	1	1.892364	-1.851109	1.184723
1	-2.611459	-1.738486	0.769978	1	1.248926	-2.294193	-0.424250
1	-1.958462	-2.074568	-0.861202	1	2.888531	-1.589491	-0.267188
1	1.318348	2.083896	-1.484527	1	0.237504	2.539112	-1.593855
1	0.561885	2.517269	0.078485	1	-0.676372	2.487265	-0.066858
1	-0.921441	-2.289056	0.557830	1	-1.447909	1.949852	-1.591126
**T_1 TS1**				**T_1 BR2**			
6	-1.545145	0.778524	0.977821	6	1.380561	-0.882218	1.003279
6	-0.487724	0.733824	-0.118953	6	0.329917	-0.676973	-0.082656
8	-0.204500	1.815960	-0.733294	8	0.158135	-1.654476	-0.927128
6	-0.217199	-0.625319	-0.887916	6	0.342286	0.722440	-0.899076
6	0.858529	-0.296597	0.122220	6	-0.878807	0.291501	0.149524
6	0.883975	-1.023076	1.404939	6	-0.908550	1.108929	1.352681
1	-1.735013	-0.182695	1.460002	1	2.314375	-1.224807	0.545835
1	-2.484744	1.133151	0.539061	1	1.579644	0.013404	1.595376
1	0.060130	-0.383971	-1.913362	1	0.048962	0.532328	-1.929144
6	-1.301724	-1.752289	-0.816603	6	1.677575	1.669407	-0.843640
6	2.225510	0.388717	-0.236375	6	-2.365226	-0.362419	-0.160331
1	0.369030	-0.678825	2.295166	1	-0.022022	1.584105	1.756491
1	1.563541	-1.865711	1.514559	1	-1.860132	1.371669	1.801954
1	-0.944357	-2.637073	-1.355451	1	2.478969	1.110070	-1.332835
1	-1.247613	1.502675	1.744257	1	1.032402	-1.665038	1.686431
1	2.134018	0.983878	-1.146455	1	-2.599449	-0.964521	0.720229
1	2.995646	-0.380013	-0.373583	1	-3.105498	0.431909	-0.282962
1	-1.526829	-2.049267	0.210406	1	1.471878	2.586110	-1.403164
1	-2.217428	-1.397883	-1.298891	1	1.969700	1.922529	0.176947
1	2.523704	1.040567	0.588747	1	-2.329041	-0.996503	-1.045923
**T_1 TS2**				**T_1 TS2B**			
6	1.360367	-0.996752	0.963260	6	-0.993339	0.747989	1.511786
6	0.259236	-0.791788	-0.069033	6	-0.291927	0.710350	0.147820
8	0.051690	-1.690668	-0.932738	8	-0.225932	1.825648	-0.525469
6	0.321180	0.817013	-0.892281	6	-0.677695	-0.478251	-0.888107
6	-0.821206	0.318355	0.137995	6	0.828848	-0.349080	-0.128000
6	-0.824247	1.082526	1.390862	6	1.035041	-1.402956	0.845230
1	2.235904	-1.436088	0.475917	1	-0.370749	0.301820	2.293884

1	1.654650	-0.086882	1.489956	1	-1.172094	1.797853	1.757188
1	0.056103	0.610037	-1.926767	1	-0.620617	-0.087798	-1.901543
6	1.697906	1.679072	-0.790412	6	-2.074403	-1.327419	-0.675063
6	-2.350978	-0.245566	-0.184764	6	2.279935	0.269976	-0.662939
1	0.069150	1.551241	1.787662	1	0.211608	-1.871380	1.373216
1	-1.759472	1.282325	1.901990	1	2.018911	-1.842247	0.969413
1	2.490622	1.084700	-1.252259	1	-2.137026	-2.075874	-1.469125
1	0.998711	-1.712187	1.712181	1	-1.960989	0.234858	1.515519
1	-2.619078	-0.842198	0.689591	1	2.112313	1.074072	-1.379042
1	-3.044359	0.590919	-0.300891	1	2.761938	0.660807	0.235462
1	1.558979	2.603531	-1.360012	1	-2.120458	-1.819719	0.298056
1	1.968354	1.926058	0.237751	1	-2.892830	-0.609769	-0.773564
1	-2.340643	-0.871951	-1.075959	1	2.883838	-0.525695	-1.104934
**T_1 BR3**				**T_1 BR3B**			
6	-1.513966	-1.087671	0.749118	6	-0.513943	-0.693356	1.303449
6	-1.063209	-0.286527	-0.460261	6	-0.216442	-0.135451	-0.144505
8	-1.489117	-0.529432	-1.572116	8	-0.251292	-1.292933	-0.901012
6	0.003742	0.854816	-0.228808	6	-1.335113	0.837802	-0.576922
6	1.347031	-0.007159	-0.299925	6	1.236647	0.472346	-0.120338
6	2.048944	-1.035994	0.733121	6	1.386156	1.794263	0.013681
6	-0.432644	2.041346	-1.365425	1	-0.545196	0.158054	1.988241
6	-0.110548	1.488548	1.114386	1	-1.472951	-1.216557	1.316489
1	-2.213407	-0.481419	1.338649	1	-1.027546	1.552730	-1.337853
1	-2.023814	-1.994899	0.416345	6	-2.851195	0.689814	-0.387549
1	-0.678803	-1.339341	1.409051	6	2.484622	-0.441784	-0.198422
1	1.730469	-0.121758	-1.313869	1	2.371892	2.250873	0.063901
1	3.131332	-0.949988	0.598486	1	0.538070	2.468848	0.086788
1	1.798745	-0.820913	1.775900	1	-3.339569	1.642289	-0.614164
1	1.748785	-2.065099	0.499141	1	0.272924	-1.385047	1.611043
1	0.279945	2.867692	-1.326445	1	-3.267669	-0.071387	-1.065725
1	-0.477045	1.599557	-2.360664	1	-3.122892	0.395940	0.633249
1	-1.425235	2.379397	-1.062697	1	2.499262	-1.005200	-1.137344
1	-0.899194	2.207209	1.318786	1	2.494694	-1.178442	0.614267
1	0.591374	1.274606	1.912740	1	3.388101	0.168138	-0.128200

References:

(S1) Frutos, L. M.; Sancho, U.; Castaño, O., *J. Phys. Chem. A* **2005**, *109*, 2993–2995.

(S2) Wilsey, S., et al. *J. Am. Chem. Soc.* **1996**, *118*, 176-184.

(S3) Ribas-arino, J. & Marx, D. *Chem Rev,* **2012**, 112**,** 5412-87.

(S4) Wolinski, K. & Baker, J. *Molecular Physics,* **2009**, 107**,** 2403-2417.

(S5) Verlet, L., *J. Physical Review.* **1967***, 159*, 98–103.

(S6) Neese, F. *Rev. Comput. Mol. Sci.* **2012**, *2*, 73–78.
